# Behavioral and Physiological Response of *Eucosma giganteana* to Semiochemicals From Conspecifics and *Silphium integrifolium*

**DOI:** 10.1002/ece3.74476

**Published:** 2026-10-28

**Authors:** Hazel Scribner, Jacqueline Maille, Ebony G. Murrell, Matthew Hetherington, Erin D. Scully, Kun Yan Zhu, William R. Morrison

**Affiliations:** ^1^ Department of Entomology Kansas State University Manhattan Kansas USA; ^2^ The Land Institute Salina Kansas USA; ^3^ USDA, Agricultural Research Service Center for Grain and Animal Health Research Manhattan Kansas USA

**Keywords:** electroantennogram, *Eucosma giganteana*, flight mill, semiochemical, *Silphium integrifolium*

## Abstract

*Eucosma giganteana* is a specialist herbivore on *Silphium* spp. and a major limiting factor to the commercialization of *Silphium integrifolium* , a perennial oilseed crop. Although it is known that (*E*)‐8‐dodecenyl acetate works as an attractant to *E. giganteana*, it is unknown whether it responds in a concentration‐dependent manner, and whether this cue works throughout the season. Also unknown is whether these moths behaviorally or physiologically respond to other important semiochemicals, such as those emitted by conspecifics or its host. This information is important for developing volatiles for use in behaviorally‐based management of *E. giganteana.* To address our questions, we performed field trapping with traps baited with (*E*)‐8‐dodecenyl acetate, characterized headspace emissions from *E. giganteana*, and used EAG and flight mill analysis to assess response to (*E*)‐8‐dodecenyl acetate and volatiles derived from *S. integrifolium* and conspecifics. Higher loadings of (*E*)‐8‐dodecenyl acetate in the field trapping studies conducted in 2024 resulted in increased captures of *E. giganteana* moths on sticky traps, which successfully monitored *E. giganteana* through their full flight period. EAG showed antennal depolarizations, which confirmed *E. giganteana* perceive (*E*)‐8‐dodecenyl acetate. In addition, female *E. giganteana* perceive volatiles emitted by *S. integrifolium* whereas males did not. Despite eliciting clear antennal responses and showing success in field monitoring, (*E*)‐8‐dodecenyl acetate did not influence flight behavior during the flight mill experiments under the tested conditions. Altogether this study shows (*E*)‐8‐dodecenyl acetate successfully monitored *E. giganteana* through the field season; more work is required to deploy it in behaviorally‐based management IPM tactics.

## Graphical abstract

There was an increase in attraction by *Eucosma giganteana* to (*E*)‐8‐dodecenyl acetate in the field, which also elicited a higher antennal response in the laboratory. Female response to its host plant was high, but it was absent in males. Altogether, this study lays the foundation for deploying (*E*)‐8‐dodecenyl acetate in behaviorally‐based management IPM tactics.

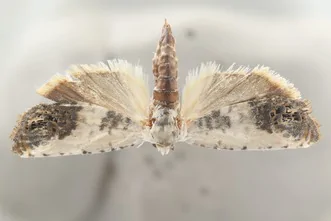

## 1 Introduction

*Silphium integrifolium* Michx is a native perennial oilseed crop currently under domestication and commercialization at the Land Institute in Salina, KS (Van Tassel et al. 2017). As a highly drought‐tolerant crop, it represents a great candidate for improving the resilience of agricultural systems to climate change (Price et al. 2022; Reinert et al. 2019; Vilela et al. 2020). A major limiting factor of *S. integrifolium* domestication is its specialist pest, *Eucosma giganteana* (Riley) (Lepidoptera: Tortricidae) (Vilela et al. 2018). These moths are apparently univoltine, and the adults fly for 1–2 months. The larvae are internal feeders on the flower heads and root crowns of *S. integrifolium* , reducing the seed set and biomass of the plant, respectively (Johnson et al. 2019; Prasifka et al. 2017). Because of their internal feeding, there is limited time to treat the larvae, making pest management difficult. Typically, *E. giganteana* infestations are treated with foliar applications of broad‐spectrum insecticides, once their presence has been detected (Murrell et al. 2023). However, optimizing the timing of insecticide applications remains challenging.

Pest population monitoring is an important aspect of any integrated pest management strategy (Miller et al. 2015). Successful monitoring generally provides insight into when the pest is present and the size of the pest population and can often be linked to a threshold for implementing pest suppression tactics. In addition, monitoring can often be paired with other decision‐support tools such as growing degree‐day models (Scribner et al. 2025). Sex pheromones have been used for monitoring and management of tortricid pests in many agricultural systems (Frerot et al. 1979; Hung et al. 2001; Knight et al. 2017). While the sex pheromone of *E. giganteana* has not yet been identified, prior work has identified the compound (*E*)‐8‐dodecenyl acetate as a general attractant for *E. giganteana* adults during flight (Ruiz et al. 2022). When combined with a clear sticky card, low concentrations of (*E*)‐8‐dodecenyl have been found to be an effective lure for monitoring *E. giganteana*. However, little is known about its prospects for use in mating disruption, or other behaviorally‐based management approaches (Morrison III et al. 2021). There has been much interest in developing a behaviorally‐based management program for *E. giganteana* in order to reduce the reliance on insecticide inputs. Currently, little is known about the chemical ecology of this pest, including how other semiochemicals *E. giganteana* encounters within its environment and affect behavioral response.

Plant volatile emissions mediate host plant selection in herbivorous insects (Schoonhoven et al. 2005). Host kairomones are often attractive to both males and females and may complement sex‐specific attractants. For example, the addition of (2E, *4*Z)‐2, 4‐decadienoate (pear ester) to pheromone lures increases male and female coddling moth capture compared to traps baited with pheromones alone (Knight et al. 2005). However, few studies have characterized the volatile emissions of *S. integrifolium* and none have investigated *E. giganteana* behavioral responses to host volatiles. The only prior work found terpenoids were upregulated in *S. integrifolium* plants when intercropped with wheat and when herbivory was induced (Fyie et al. 2025).

One method to quickly determine whether pests can detect volatiles from complex blends is to use gas chromatography coupled with electroantennography detection (GC‐EAD) or electroantennography (EAG). EAG is a technique that uses insect antennae as a biosensor for identifying physiologically active compounds (Brezolin et al. 2018). It has been used to evaluate the physiological response of many species, including tortricid species, to semiochemicals (Casado et al. 2006; Fu et al. 2022; McNair et al. 1999). As such, it is a highly useful tool for answering questions about what a particular species can detect in its environment.

An important component of behavioral response to semiochemicals is how they may affect flight behavior. Flight capacity in the laboratory is a key measure and important for understanding the dispersal capabilities of both field and stored product pests (Abshire et al. 2024; Jones et al. 2016; Naranjo 2019). Both static tethering and flight mills have been used in the past to measure dispersive capabilities of numerous insect species (Naranjo 2019). Critically, pheromones, kairomones, and food cues have been shown to influence the flight distance and velocity of individuals of other moth pests (Abshire et al. 2024). Dispersal capabilities for multiple other tortricid species have been examined, including the oriental fruit moth (*Cydia molesta* Busck) and the codling moth (*Cydia pomonella* L.) (Hughes et al. 2004; Matveev et al. 2017; Schumacher et al. 1997). In prior work, Ruiz et al. (2022) examined how attractants found in congener species, including a low concentration of (*E*)‐8‐dodecenyl acetate, affected the flight capacity of *E. giganteana*. The presence of (*E*)‐8‐dodecenyl acetate had arresting properties and decreased flight distance by *E. giganteana* on the flight mill by 78 to 80% compared to controls without the stimulus. However, it is unknown whether higher concentrations of this compound elicit more extreme behavioral effects.

Together, understanding the headspace emitted by conspecifics and their host plant, as well as the behavioral and physiological responses by *E. giganteana* to those semiochemicals, will help lay the foundation for a behaviorally‐based management strategy for this species (Morrison III et al. 2021). These strategies include mating disruption and attract‐and‐kill, which may be ideally suited for a pest such as *E. giganteana* with generally low to intermediate population size. Thus, our aims were to examine the effects of different dosages of (*E*)‐8‐dodecenyl acetate on *E. giganteana* attraction over the course of a season using a (1) linear set of concentrations, or (2) an exponential set of concentrations in 2023 and 2024. Additionally, we quantified male and female *E. giganteana* reaction to semiochemicals from conspecifics and its host species, *S. integrifolium* , by (1) characterizing headspace emissions, (2) examining male and female *E. giganteana* flight capacity on a tethered flight mill, and (3) assessing the physiological response of *E. giganteana* of both sexes using EAG.

## 2 Materials and Methods

### 2.1 Trapping in 2023 With a Linear Set of (*E*)‐8‐Dodecenyl Acetate Loadings

Field trapping was done according to the methodology in Ruiz et al. (2022) and Scribner et al. (2025). The fields were located in North‐Central Kansas at the Land Institute near Salina, KS (Table 1). No pesticides were applied to these fields during the experiment in 2023. Starting the first week of June, six transects were set out, two in each *S. integrifolium* field. Each transect contained seven 30.4 × 30.4 cm sticky card traps (Alpha Scents, Canby, OR, USA) affixed to the top of a 1.27 cm diameter, three‐foot in length PVC pole that was hammered into the ground until sturdy. The cards were affixed using a 271 cm long sticky card ring holder (Olson Products Inc., Medina, OH, USA) that was bent to a 90° angle and placed inside the PVC pipe. Two large binder clips were also used to anchor the sticky card to its card holder.

**TABLE 1:** Summary of field sites used for trapping *Eucosma giganteana* in 2023 and 2024 in Salina, KS.

Field ID	Year	Latitude	Longitude	Area (ha)	Insecticide application?
A	2023	38.771	−97.570	0.226	No
B1	2023	38.773	−97.592	0.152	No
B2	2023	38.770	−97.593	0.152	No
B3	2024	38.772	−97.593	0.168	Yes
C	2024	38.770	−97.599	0.557	No
D	2024	38.698	−97.575	0.141	Adjacent

The seven sticky traps in each transect were spaced with 10 m between them around the perimeter of the field. In 2023, traps within each transect were baited with lures containing linearly increasing concentrations of (*E*)‐8‐dodecenyl acetate, including either a solvent‐only control (e.g., 50 μL of acetone), a low loading with 51 μg, or a doubled loading with 101 μg of (*E*)‐8‐dodecenyl acetate (Alfa Chemistry, Ronkonkoma, NY, USA) (Table S2). Each loading was the final amount in 50 μL of acetone (Wheaton, DWK Life Sciences, Millville, NJ, USA) using stock solutions of 1.15 μg/mL (low), 2.30 μg/mL (doubled) (*E*)‐8‐dodecenyl acetate, or neat solvent (solvent only), which was added to the LDPE dropping bottle with a pipette. Within each transect, there were 2–3 control, 2–3 low‐loaded, and 2–3 doubled‐loaded traps whose placement and extra third treatment were randomized at the outset of the season (Table S1). Transects with two or three of each treatment rotated during lure changes through the season. The first 2 weeks in 2023 contained only control traps because they were deployed technically prior to the beginning of flight for *E. giganteana*, and (*E*)‐8‐dodecenyl acetate was only available in smaller quantities at the start of the year. The clear sticky card traps were collected and replaced biweekly on 16 June and 28 June until the first *E. giganteana* adult was captured, which was triggered by the capture of an *E. giganteana* adult in a UV light trap on the same farm, then all treatments were set out and the 42 traps were replaced weekly. Because the two samples leading up to the flight period were excluded from the statistical analysis, no correction to trapping days for each treatment was required. The lures were replaced once every 2 weeks and their position within the transect was rotated by one spot down the transect after each lure change. Each lure was used in each position twice over the course of the season. The headspace release rate has already been confirmed over a 14 days period as 28.4 ng/h when initially loaded in an LDPE dropping bottle with 50 μg of (*E*)‐8‐dodecenyl acetate (Ruiz et al. 2022).

When collected, the sticky cards were held in a 7.6 L (=2 gal) labeled Ziploc bag transported back to USDA‐ARS. All collected sticky traps were placed in a freezer for approximately 24 h. The total number of *E. giganteana* per trap and their distance from the lure in millimeters were recorded. In addition, the number of nontarget lepidoptera was recorded on each trap. Individual *E. giganteana* and nontarget lepidoptera were only counted if more than half of the specimen was remaining on the sticky trap at the time of counting to ensure positive identification.

### 2.2 Trapping in 2024 With an Exponential Set of Loadings of (*E*)‐8‐Dodecenyl Acetate

Field trapping in 2024 was conducted similarly to that in 2023 with the following modifications. Three different fields located at the Land Institute were used (Table 1). Plots that reached a threshold of > 10% infestation in *S. integrifolium* plants by *E. giganteana* larvae were treated with Prevathon (A.I.: chlorantraniliprole, which is a diamide). However, this only occurred in one field near the end of data collection for monitoring for *E. giganteana* in our study, so likely had a negligible effect on our data. Three transects were deployed in each of the three fields. Each transect contained four traps for a total of 36 traps deployed each week. The traps were assembled similarly to those used in 2023, but a hand‐made sticky card was used instead of a manufactured one to improve captures. These sticky cards were made of a laminated 21.6 × 27.9 cm (=8.5 by 11 in) piece of white cardstock paper (Astrobright, Neenah, WI, USA) coated on both sides with TAD all‐weather adhesive (Trécé Adhesives Division, Adair, OK, USA). The sticky sides were covered with wax paper for ease of travel. Additionally, the sticky cards had a 20‐gauge chicken wire cage placed over them in the field to try to prevent the capture of birds and other large nontarget species on the traps. Traps in 2024 were baited with an exponential set of loadings of (*E*)‐8‐dodecenyl acetate. In each transect, there was a solvent only control (50 μL of acetone), 50 μL of a low loading equivalent to the 2023 treatment (with a loading of 51 μg of (*E*)‐8‐dodecenyl acetate), a medium loading with 691.6 μg of (*E*)‐8‐dodecenyl acetate, and a high loading with 5113 μg of (*E*)‐8‐dodecenyl acetate (Table S2). The loadings conformed to the fitted line *y* = 0.25 · *A*_Appl_ · *e* ^*x*^ where *A*_Appl_ is a fixed constant representing the amount added (in μL), *x* represents the stock concentration (in μg/μL), and *y* represents the loading mass of compound (in μg). Each loading was the final amount in 50 μL of acetone (Wheaton, DWK Life Sciences, Millville, NJ, USA) using stock solutions of 1.15 μg/mL (low), 15.7 μg/mL (medium), and 116 μg/mL (high) of (*E*)‐8‐dodecenyl acetate, or neat solvent (solvent only), which was added in the LDPE dropping bottle with a pipette. The traps were replaced weekly, and the lures were replaced biweekly, as well as rotated in position, within the transect. Treatments were initially randomized at the outset of the season and then rotated at each lure change. Thus, each lure was in each position within the transect twice over the course of the season.

### 2.3 *E. giganteana* and *S. integrifolium* Collections From the Field

*Eucosma giganteana* cannot yet be reared successfully in the laboratory, thus we sourced all specimens from the field. Adult *E. giganteana* individuals were carefully captured by hand in one of the fields planted with *S. integrifolium* at the Land Institute (38.769622, −97.598576) between 22:00 and 24:00 five times a week from June to August 2024. Moths were immediately sexed and individually placed in small deli cups with appropriate labels. They were brought back to the USDA‐ARS Center for Grain and Animal Health (39.1955486, −96.5987334) for the experiments described below. Once in the lab but prior to use in experiments, moths were kept in a low traffic environment at approximately 23°C s 0.1°C and 16:8 L:D photoperiod. Importantly, no lures were used to capture insects to avoid biasing the results of the experiments below. *S. integrifolium* flower heads were cut 1 cm below the flower and brought back on a weekly basis during the same timeframe and stored at 4°C until needed for experiments. Flower heads were never more than 4 days old prior to use.

### 2.4 Headspace Characterization

Headspace was collected from the following treatments: 10 *E. giganteana* male moths only (*n* = 5 replicates), 10 *E. giganteana* female moths only (*n* = 7), an even mix of male and female moths (5:5) (*n* = 5 replicates), flower cuttings of *S. integrifolium* (*n* = 9), and a blank control (*n* = 8). For the *E. giganteana* treatments, only live, healthy adult moths that were collected within 5 days were used. For the *S. integrifolium* collections, approximately 25 g of flower heads were cut the same week as insect collections were used.

For each treatment, *E. giganteana* or *S. integrifolium* was placed in a clean 100 mL beaker. To prevent moth escapees, a metal mesh top was constructed and affixed to the opening of the beaker. The beaker was then placed in one of eight 500 mL glass headspace collection containers with a PTFE septum and lid. A Porapak Q volatile collection trap (VCT) was inserted in the output end. The VCT consisted of an angled drip‐tip point made of a borosilicate glass tube with a mesh (Stainless Steel #316 screen), packed with 20 mg of Porapak‐Q chemical absorbent held in place with a borosilicate glass wool plug, and followed by a PTFE Teflon compression seal (Volatile Collection System, Gainesville, FL, USA). A PTFE tube spanned from the flow meter (CADS‐4CPP, Clean Air Delivery System, Sigma Scientific LLC, Micanopy, FL, USA) to the input end of the headspace container at a flow rate of 1 L/min. Prior to that, the air was scrubbed with an activated carbon filter and was pumped in using the central air pump for the center. Samples ran for a period of 24 h. Each volatile collection trap was collected and eluted with 150 μL of dichloromethane in a fume hood into a 2 mL GC vial containing a 250 μL glass insert with polymer feet. The solvent was gently pushed through the volatile collection trap with N_2_ gas. Once all samples were collected, 1 μL of an internal standard, tetradecane (190.5 ng), was added to each of the samples. The samples were then all capped with a magnetic screw top lid and secured with PTFE tape before being placed in a freezer at −20°C until they could be run. All headspace samples were collected within 5 weeks. After each replication, the headspace collection containers were washed with methanol and then hexane. VCTs were rinsed in triplicate with 700 μL of dichloromethane.

### 2.5 Gas Chromatography Coupled With Mass Spectrometry

All headspace collection sample extracts were run on an Agilent 7890B gas chromatograph (GC) equipped with an Agilent Durabond HP‐5 column (30 m length, 0.250 mm diameter and 0.25 μm film thickness) with He as the carrier gas at a constant 1.2 mL/min flow and 40 cm/s velocity. The GC was coupled with a single‐quadrupole Agilent 5997B mass spectrometer (MS). The compounds were separated by auto‐injecting 1 μL of each sample under splitless mode into the GC–MS at room temperature (approximately 23°C). The flow rate was 1.2 mL/min. The GC program consisted of 35°C for 3 min followed by 5°C/min increases to 120°C and then 10°C/min increases to 250°C for a total run length of 33 min. After a solvent delay of 4 min, mass ranges between 50 and 550 amu were scanned. Compounds were tentatively identified by comparison of spectral data with those from the NIST 14 library and by GC retention index. The samples were normalized according to the following formula (Pk_sam_ − Pk_min_)/(Pk_max_ − Pk_min_), where Pk_sam_ is the peak area from the given sample, Pk_min_ is the global minimum peak area for the dataset, and Pk_max_ is the global max peak area for the dataset. Thus, relative peak areas were normalized by min‐max scaling, and values could vary between 0 to 1. A total of 20 certified terpene standards (Terpene Mix A, Supelco, Bellefonte, PA, USA) at 2000 μg/mL per compound in methanol were injected at 1 μL without dilution separately after the headspace samples on the same column.

### 2.6 Electroantennography of *E. giganteana*

All electroantennogram (EAG) recordings of *E. giganteana* were taken from 19:00 to 23:00 which corresponded to the peak activity period of *E. giganteana* based on prior literature (Ruiz et al. 2022). Only moths captured from the field within the past 3 days were used for recordings. The moths were knocked down in a freezer for 1–2 min. The antenna of the moth was ablated at the pedicle as close to the scape as possible, alternating between left and right antennae for each recording. Then, using electroactive gel (Spectra 360 Electrode gel, Parker Laboratories INC., Fairfield, NJ, USA), the ablated antennae were placed on an antenna holder (Syntech GmbH, Buchenbach, Germany) and covered with additional electroactive gel to ensure connection and prevent desiccation. The antennal holder was then slotted into the EAG probe (EAG Combi Probe, Syntech GmbH, Buchenbach, Germany). Once the baseline stabilized for 1–2 min on the EAG recording, a flow meter (CS‐55, Syntech GmbH, Buchenbach, Germany) was used to deliver stimuli to the antennae by puffing air at a rate of 0.3 μL/s through a Pasteur pipette. Once the flow meter was triggered, the stimulus was delivered twice with a 30 s delay before the first puff, then a 1 min delay before the second puff.

The EAG stimuli treatments included blank air, neat (e.g., high) (*E*)‐8‐dodecenyl acetate (Alfa Chemistry, Ronkonkoma, NY, USA) with 5022 μg, dichloromethane solvent control, a low loading of 6.07 μg (*E*)‐8‐dodecenyl acetate, 150 μL dichloromethane +1 μL tetradecane (190.5 ng/μL) control, female *E. giganteana* headspace (see above), *S. integrifolium* headspace (see above), and neat acetone. Six μL of each stimulus was pipetted into the center of a small filter paper (2.5 cm diameter) (Table S2), which was then folded and placed into the top of a Pasteur pipette. The stimuli were presented in the order given. Apart from the blank control which was puffed four times at the beginning of each recording and twice at the conclusion of each recording, all other treatments were only puffed twice. The Pasteur pipettes containing the treatments were replaced after an hour to ensure the antennae were supplied with a fresh odor source. The two puffs per individual were considered technical replicates, but there was a total of *n* = 15 biological replicates (e.g., antennae from separate individuals) for female moths and *n* = 17 biological replicates for male moths for each treatment. The corrected antennal depolarization was used and calculated as (Δ*A*_trt_ − Δ*A*_blank_) − (Δ*A*_DCM_ − Δ*A*_blank_), where Δ*A*_trt_ is the change in amplitude (e.g., antennal depolarization) as a result of the treatment in each replicate, Δ*A*_blank_ is the change in amplitude (e.g., antennal depolarization) as a result of blank air in each replicate, and Δ*A*_DCM_ is the change in the amplitude (e.g., antennal depolarization) as a result of the solvent only for each replicate. Thus, in figures, only five treatments are displayed since the blank and DCM solvent controls were used to standardize antennal responses.

### 2.7 Flight Mill Apparatus

The flight mill was based on the design found in Attisano et al. (2015) and Stowe et al. (2022). In short, there were 25.5 × 24.5 × 12 cm (length × width × height) plexiglass shelves stacked 4 by 4 vertically adjacent to each other. A hypodermic needle (19‐gauge nonmagnetic hypodermic steel tubing) 22 cm long comprised the flight arm assembly and was affixed to a 10 μL micropipette tube in a center pivot with hot glue and held weightless by two strong antipolar neodymium magnets ventrally and dorsally. The flight rod is horizontal with one end for a flag and the other end to hold an insect. The flag is used to interrupt the LED/PT sensor (OPB800W, Optek Technology Inc., Texas USA) which changes the analog signal from low voltage to higher voltage. In each shelf, there was a sensor that measured when the signal was broken by the revolution of the flight arm to record flight using a datalogger (DATAQ Model DI‐2108‐P data acquisition interface and software, DATAQ Instruments, Ohio, USA), and flight was logged continuously, streamed to an attached computer using customized software. The interface has +5 VDC and ground terminals for supplying power to the sensors. The hardware includes an array of screw‐terminals for connecting the wires from the 8‐LED/PT sensors. The sensors are interfaced to a PC via a USB cable. The analog signals are converted to digital signals and transferred to the computer for data collection and processing.

### 2.8 Flight Capacity of *E. giganteana* in Response to Semiochemicals

Only moths captured within 1–3 days of the start of flight mill recording were used. Ten to 12 moths were prepared at a time for the eight flight mills. The 89 mL (=3 oz) solo cups containing these moths were placed in an ice bath to partially knock them down. Individual moths were then removed, and their scales were gently brushed off their pronotum using a small silicone brush to allow better adherence of the glue. A small dollop of T‐7000 glue was applied to the blunt end of a size 2 entomology pin (Bioquip, Rancho Dominguez, CA, USA), and this was pressed against the pronotum for 1 min to allow time for the glue to dry. The pins with moths were inserted into an elevated piece of polystyrene until the moths were ready to be placed on the flight mill. Eight of the 10–12 moths were run simultaneously on separate, individual flight mills, choosing moths with no defects in attachment and with individuals that were more active (after Attisano et al. 2015). Moths that could not properly beat their wings due to the glue or wing placement were not used on the flight mills. The tip of the pin of each moth was inserted into the arms of the flight mill and positioned to fly counterclockwise. Each moth was encouraged to fly by initially gently blowing on its abdomen for ~1 s to make sure the arm with the moth on it could pass through the sensor. Once all moths were set up and vetted, moths were given the opportunity to fly in a 24 h period. After the end, the moths were removed from the flight mill arms, their condition was noted, they were weighed on a microbalance (QUINTIX2102‐1S, Sartorius AG, Göttingen, Germany), and then they were frozen. The moths were weighed to evaluate whether size affects flight capacity.

For each round of the flight mill, each sex was run independently (blocked) to determine if flight capacity was altered in the presence of the other sex. An additional block included both sexes being run, which consisted of four male and four female moths running simultaneously. The semiochemical treatments included a low loading of (*E*)‐8‐dodecenyl acetate with 25.3 μg of (*E*)‐8‐dodecenyl acetate and a high loading of 2555 μg of (*E*)‐8‐dodecenyl acetate using the same stock solution concentrations for the LDPE dropping bottle as in the field baiting experiment for 2024 (Table S2). For all semiochemical treatments, a total of 25 μL was pipetted onto a piece of filter paper (10 cm diameter) and placed in a Petri dish (100 × 15 mm diameter: height). The Petri dish was left uncovered in front of and centered adjacent to the flight mill. In total, there were two blocks of eight moths used for each of the treatments, so *n* = 16 behavioral replicates for each treatment combination of sex and semiochemical.

### 2.9 Data Analyses

To analyze the field trapping data in 2023 and 2024, a generalized linear mixed model (GLMM) with a quasipoisson distribution was run to account for overdispersion in the dataset using the *lme4* package (Bates et al. 2015). A log‐link function was used because of the ease in interpreting the resulting coefficients. Response variables included *E. giganteana* or nontarget Lepidoptera captures. Separate models were run for each year and response because of differences by year in experimental design; thus, these were treated independently. Models were otherwise identical for each response and year. Random variables included block and date. Treatment (ctrl, low, and doubled loading in 2023 or ctrl, low, medium, and high loading in 2024) was used as a fixed, explanatory variable. F statistics were estimated with restricted maximum likelihood (REML). To assess fit, residual by predicted plots and histograms were inspected, and a fitted model based on Poisson, quasipoisson, and negative binomial were graphically assessed. Upon a significant result from the model, Tukey HSD was used for multiple comparisons. R software was used for all analyses (R Core Team, 2024), with *α* = 0.05, except where noted.

To analyze the headspace collected from the odor treatments, raw chromatograms were deconvoluted and integrated in MassHunter Unknowns Analysis (Agilent Technologies Inc., Santa Clara, CA, USA) after GC–MS. Peaks with a height < 0.3% the height of the tallest peak were excluded from the exported peak table. Compounds were aligned across samples by obtaining the underlying *m*/z ratio data from publicly linked repositories, including Pubchem, and flexibly aligning compounds based on structural matches and retention time, and normalized emission rates were calculated using the R package *uafR* (Stratton et al. 2024). Volatile compounds appearing in fewer than two samples were discarded, as these represent transient background volatiles in the general vicinity of headspace collection but are not informative of differences among the treatments. Additionally, siloxane (including *m*/*z* ions 73, 147, 207, 221, 281, and 355) and phthalate (including *m*/*z* ions 149, 167, and 104) peaks were removed as these represent common contaminants in GC–MS analysis. All other volatiles exceeding a 70% match to the reference NIST14 library were retained in the analysis. Pairwise Bray–Curtis similarities were calculated between headspace samples, and nonmetric multi‐dimensional scaling (NMDS) was used to visualize the scaled differences in relative peak areas among treatments. A total of *n* = 1000 permutations were used for the ordination procedure. The dimensions were set to k = 3 as this provided a better resulting ordination. Stress values for the NMDS procedure were less than 0.13, indicating that good interpretation was possible. The package ggplot2 was used for plotting the NMDS (Wickham 2016). Analysis of similarity (ANOSIM) was applied to test whether the volatile profiles varied among treatments using the R package *vegan* (Oksanen et al. 2024). A total of *n* = 1000 permutations were performed for the statistical test. Following the global ANOSIM, pairwise ANOSIMs were performed using the *veganEx* package with a Holm‐Bonferroni correction to the *p*‐value (Zhonghui 2021).

Before the EAG data could be analyzed, the depolarizations following each stimulus puff were quantified for each moth's recordings using the software GC‐EAD 0.1.2.5 (Syntech, Kirchzarten, Germany). Within each recording, the average depolarization for each blank in each replicate across blank puffs was subtracted from all the other stimulus puffs to remove the baseline *E. giganteana* reaction. Similarly, the DCM and/or the DCM plus tetradecane depolarizations were subtracted from all treatments that included DCM and/or DCM plus tetradecane in the solutions. This value left us the actual depolarization for only the treatments of interest. A linear mixed model (LMM) was used to analyze the corrected depolarization, with repeated measures on moth antennae coded as a random variable. Fixed, explanatory variables included semiochemical treatment, puff number (1 or 2), sex (male or female), and each two‐way and three‐way interaction. The LMM was implemented using the *lme4* package (Bates et al. 2015), and *p*‐values were estimated based on a chi‐square distribution. Upon a significant result from the model, we used Tukey HSD for multiple comparisons implemented with *emmeans* and the function *cld* (Lenth and Piaskowski 2026).

Before analysis, flight mill data was first vetted to eliminate all flights that were clockwise, indicating that the moth was drifting and not flying, as well as those with no recorded directionality, which indicated that the moth's flag was stuck near the sensor. Then, distance flown was calculated based on the circumference of the circle made with the flight mill arm (58.975 cm) and the instantaneous velocity was calculated by dividing that set distance by the time it took to complete the rotation. An initial model ruled out that the weight of moths was not significantly different among the semiochemical treatments (df = 3, 98; *F* = 0.30; *p* = 0.83; Figure S1), and the interaction between weight and semiochemical treatment did not significantly affect distance flown (df = 3, 94; *F* = 0.93; *p* = 0.43). The final model analyzed flight distance using a general linear model with flight distance or velocity as the response variable. The fixed, explanatory variable included sex (male or female), date, semiochemical treatment (control, low, or high (*E*)‐8‐dodecenyl acetate), weight of moths as a covariate, as well as the 2‐way interaction between sex and semiochemical treatment. In a model for a subset of data only containing the controls, the effect of the presence of single sex and dual sex (four males and four females) was investigated as a fixed explanatory variable. Residuals and histograms were inspected to ensure assumptions of normality and homogenous variance in both cases, and where there were deviations a square root or log transformation was used, which corrected deviations. Upon a significant result from the model, Tukey HSD was used for multiple comparisons.

## 3 Results

### 3.1 *E. giganteana* Captures in 2023 and 2024

There was a significant difference in *E. giganteana* captures among the linear loadings (Table 2). The low and doubled loading of (*E*)‐8‐dodecenyl acetate consistently captured more *E. giganteana* over the course of the season (Figure 1). Because of the varying treatments and trap design, each year was analyzed separately. Most (> 98%) trap locations were independent from each other in 2023 (Table S3). In 2024, the high loading of (*E*)‐8‐dodecenyl acetate consistently captured more *E. giganteana* than the other loadings (Figure 1). There was also a clear peak in captures during the week of June 28th through July 27th in both years. Additionally, most of the trap locations in 2024 (> 97%) were independent from each other in 2024 (Table S4).

**TABLE 2:** Statistical summary of a generalized linear mixed model based on a quasi‐Poisson distribution to account for overdispersion when evaluating *Eucosma giganteana* and nontarget Lepidoptera where treatment is the loading of (*E*)‐8‐dodecenyl acetate.

Model^a^, ^b^	df	*E. giganteana*		df	Nontargets	
		*F*	*p*		*F*	*p*
2023	2	7.58	0.001	2	1.05	0.36
2024	3	13.39	0.001	3	0.251	0.86

^a^ 2023 model residual df = 360.

^b^ 2024 model residual df = 307.

**FIGURE 1:**
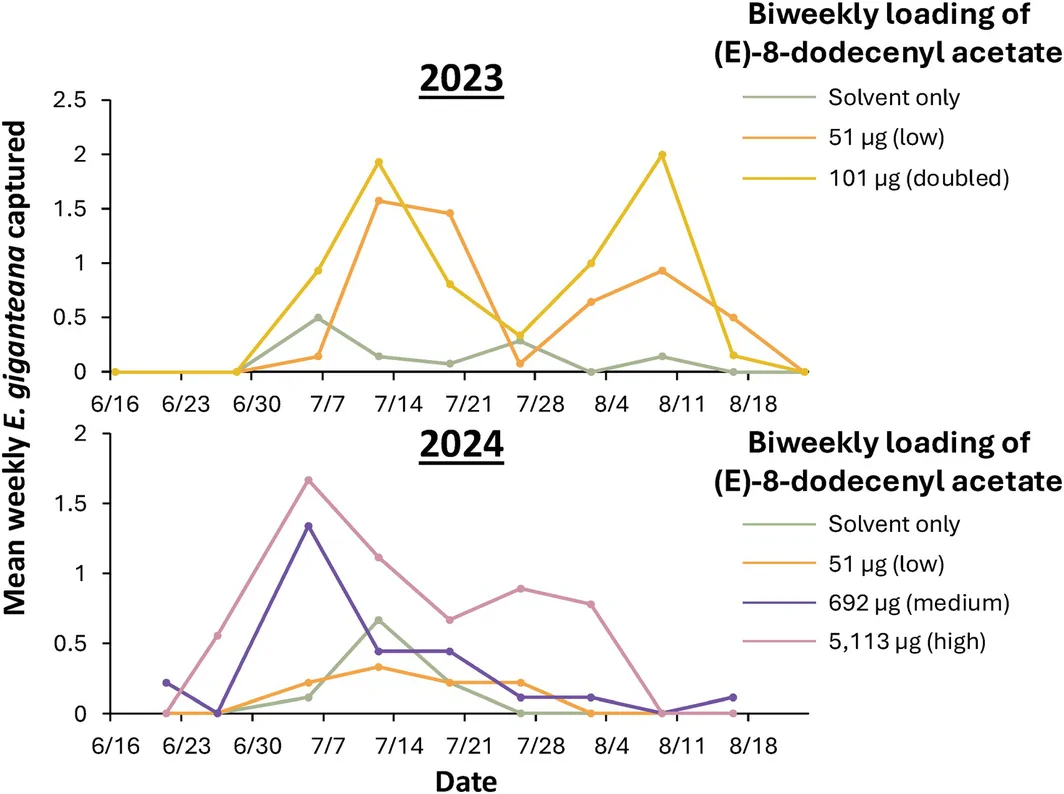
Mean weekly captures of *Eucosma giganteana* conducted in 2023 (top panel) on clear sticky cards baited with linear loadings of (*E*)‐8‐dodecenyl acetate from June through August (*n* = 12–18 traps per treatment each week) and 2024 (bottom panel) on white sticky cards baited with exponential loadings of (*E*)‐8‐dodecenyl acetate from June through August (*n* = 9 traps per treatment each week).

During trapping in 2023, the low (51 μg) and doubled loadings (101 μg) of (*E*)‐8‐dodecenyl acetate captured significantly more *E. giganteana* than the control (Table 2, Figure 2). In particular, those loadings captured 4‐ and 6‐fold more individuals, respectively, than the control. However, there was no significant difference between the low and doubled loadings of (*E*)‐8‐dodecenyl acetate. In 2024, *E. giganteana* captures were also affected by the loadings (Table 2, Figure 2). More specifically, there was a 3‐ and 6‐fold higher capture of *E. giganteana* in the medium‐ (692 μg) and high‐loaded (5113 μg) traps, respectively, compared to the low loadings (51 μg) and unbaited controls. The low loading captured a similar number of *E. giganteana* as the unbaited control in 2024 (Figure 2). However, there was not a proportional increase in captures among increasing loadings.

**FIGURE 2:**
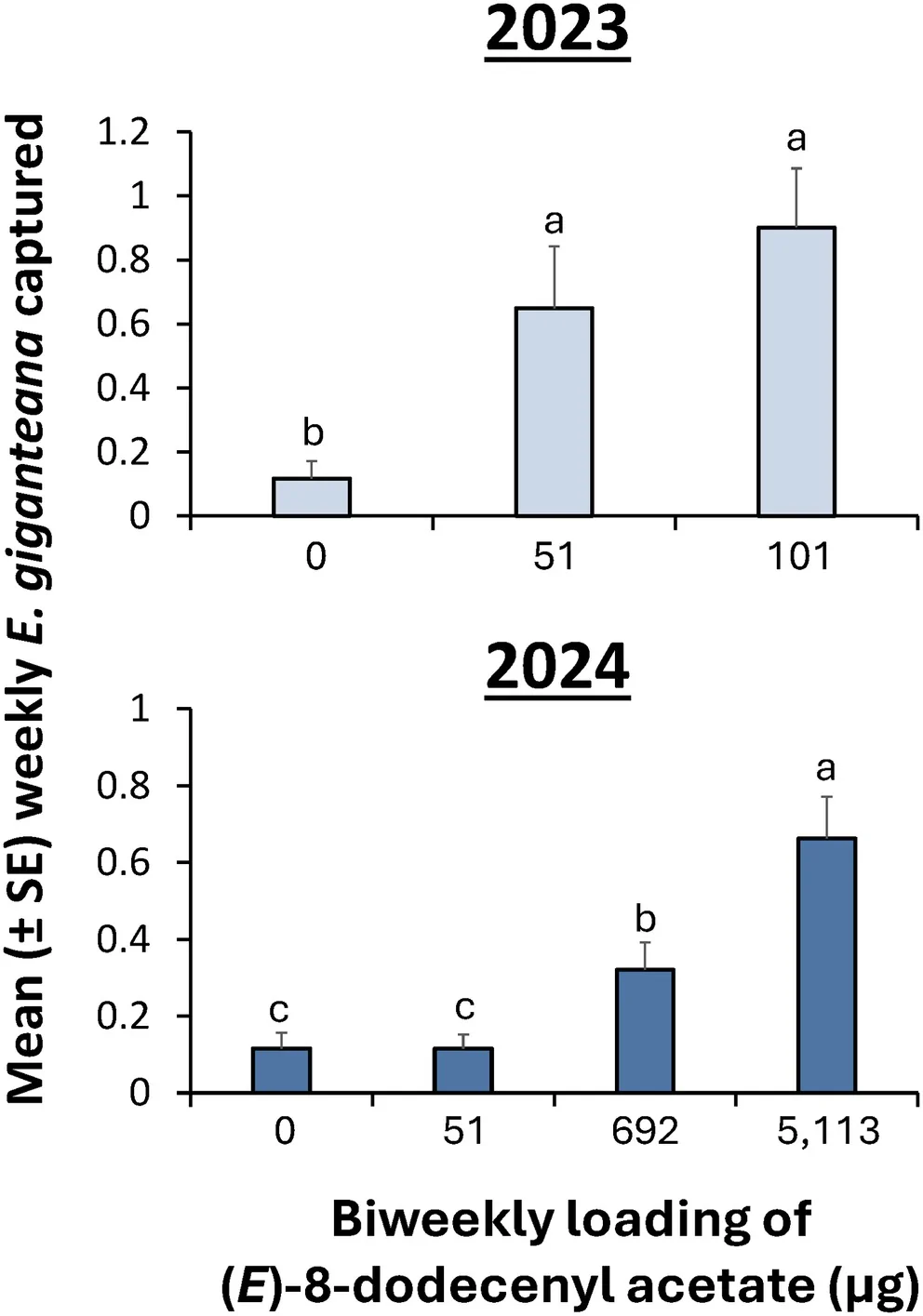
Season‐long weekly mean captures (± SE) of *Eucosma giganteana* for trapping conducted with a linear loading of (*E*)‐8‐dodecenyl acetate on clear sticky cards in 2023 (top panel) (*n* = 112–137 traps per treatment) and an exponential series of loadings on a biweekly basis on white sticky cards in 2024 (bottom panel) (*n* = 78–79 traps per treatment) from June through August. Bars with shared letters are not significantly different from each other (Tukey HSD, *α* = 0.05).

In 2023, we also measured the distance the *E. giganteana* individuals were found on the trap from where the lure was placed. We did not find a significant difference between treatments in the location of *E. giganteana* on the trap (Log likelihood = 1.51; *p* = 0.68) (Figure 3). However, almost all the moths were captured on the inside fold of the doubled‐over sticky card where the lure was deployed, and not on the external‐facing surface.

**FIGURE 3:**
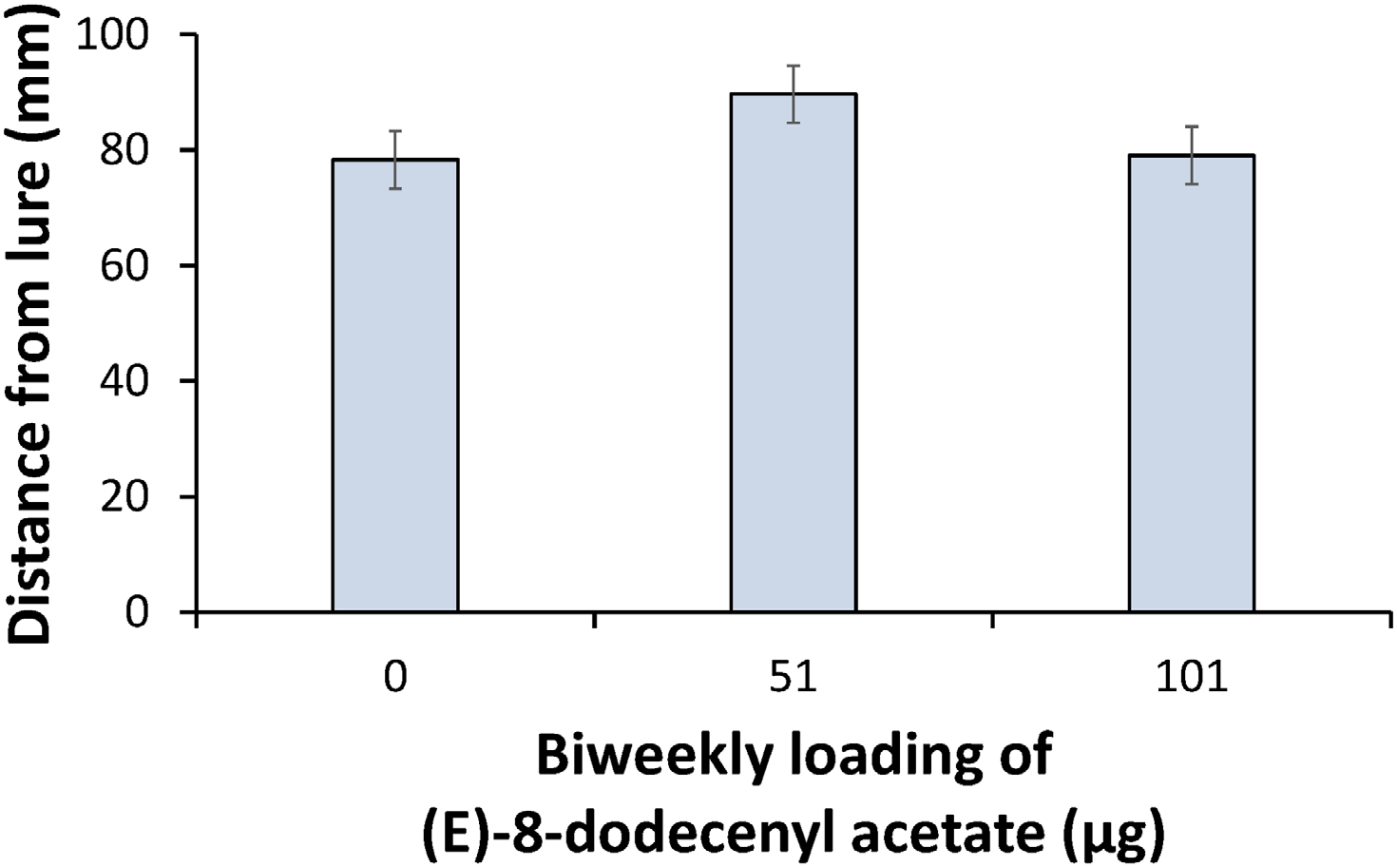
Distance (± SE) from the lure (mm) that *Eucosma giganteana* individuals were stuck on the sticky card during 2023 field trapping. There were no significant differences between the control, 51 μg, or 101 μg lure loadings, so letters were omitted from the graph (Tukey HSD, *α* = 0.05).

### 3.2 Nontarget Lepidoptera Captures in 2023 and 2024

The loadings of (*E*)‐8‐dodecenyl acetate did not significantly affect captures of nontarget Lepidoptera in 2023 or 2024 (Table 2; Figures 4 and 5). Notably, captures of nontarget were not elevated beyond that found for the unbaited control traps in either year, suggesting other taxa do not respond to (*E*)‐8‐dodecenyl acetate.

**FIGURE 4:**
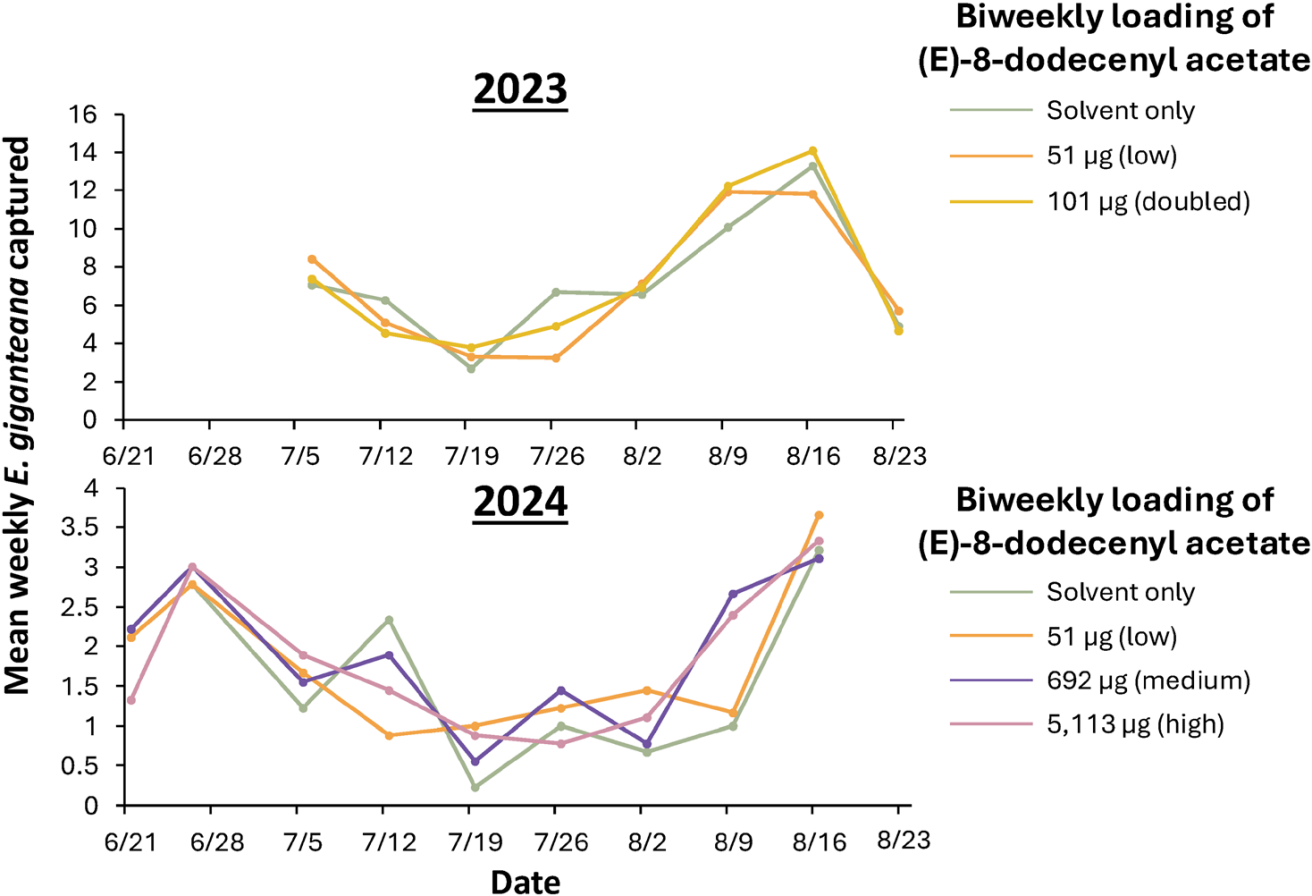
Weekly captures of nontarget Lepidoptera conducted in 2023 (top panel) on clear sticky cards baited with linear loadings of (*E*)‐8‐dodecenyl acetate from June through August (*n* = 12–18 traps per treatment each week) and 2024 (bottom panel) on white sticky cards baited with exponential loadings of (*E*)‐8‐dodecenyl acetate from June through August (*n* = 9 traps per treatment each week).

**FIGURE 5:**
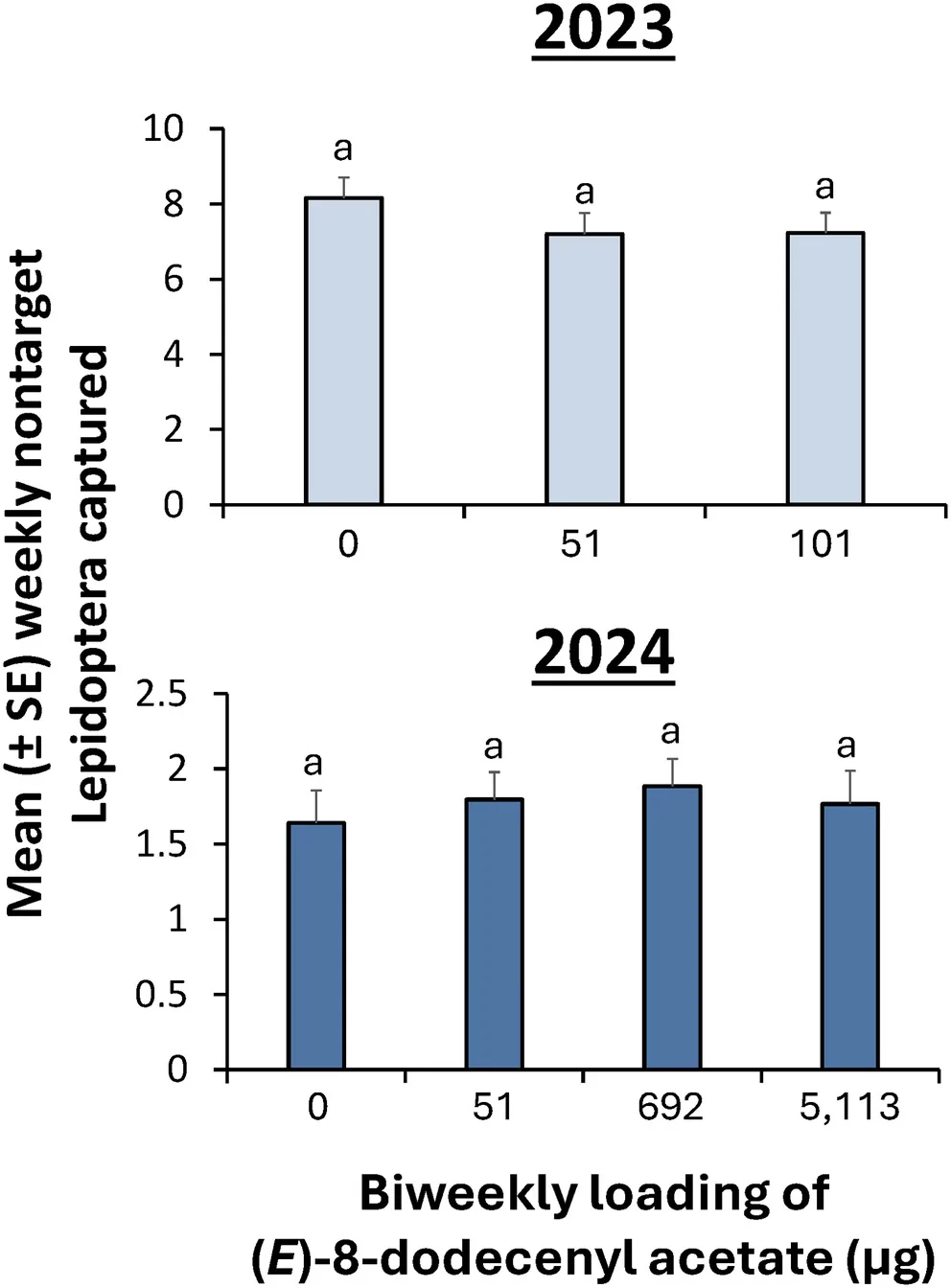
Season‐long weekly mean captures (±SE) of nontarget Lepidoptera for trapping conducted with linear loadings of (*E*)‐8‐dodecenyl acetate on clear sticky cards in 2023 (top panel) (*n* = 112–137 traps per treatment) and an exponential series of loadings of (*E*)‐8‐dodecenyl acetate on white sticky cards in 2024 (bottom panel) (*n* = 78–79 traps per treatment) from June through August by loading at the Land Institute in Salina, KS. Bars with shared letters are not significantly different from each other (Tukey HSD, *α* = 0.05).

### 3.3 Headspace Collection

There were significant differences between the headspace composition of the different treatments with the *S. integrifolium* headspace composition differing from all other treatments (Table 3; Figure 6). Scaled relative peak areas from *S. integrifolium* had 2‐, 1.8‐, 2.3‐, and 1.6‐fold more compounds than the control, female, male, and mixed cohorts of *E. giganteana* respectively (Table 4). None of the *E. giganteana* treatments differed significantly from each other or the control (Table 3). The compounds confirmed as camphene and tentatively identified as 1,4‐difluoro‐1,3‐butadiyne were relatively enriched in *S. integrifolium* headspace compared to the other treatments. Overall, the following compounds were confirmed: β‐myrcene, β‐phellandrene, β‐pinene, camphene, and limonene (Figure S2). Headspace collected from male *E. giganteana* was clustered together (Figure 6). A peak tentatively identified as carbonic chloride fluoride was relatively enriched in headspace from female *E. giganteana* compared to headspace from male *E. giganteana*, mixed *E. giganteana*, and *S. integrifolium* by 43‐, 8.6‐, and 2.3‐fold (Table 4).

**TABLE 3:** Statistical results of overall and pairwise ANOSIM tests in evaluating differences among *Eucosma giganteana* adults and *Silphium integrifolium* flower heads with bolded lines indicating a significant result.

Comparison		*R*	*p*
Overall	All	**0.202**	**0.002**
Pairwise	Ctrl vs. Female	0.0601	0.22
	Ctrl vs. Male	−0.0474	0.62
	Ctrl vs. Mix	−0.00526	0.46
	Ctrl vs. *Silphium*	**0.260**	**0.005**
	Female vs. Male	0.00829	0.49
	Female vs. Mix	−0.13	0.18
	Female vs. *Silphium*	**0486**	**0.002**
	Male vs. Mix	−0.056	0.67
	Male vs. *Silphium*	**0.498**	**0.007**
	Mix vs. *Silphium*	**0.529**	**0.001**

**FIGURE 6:**
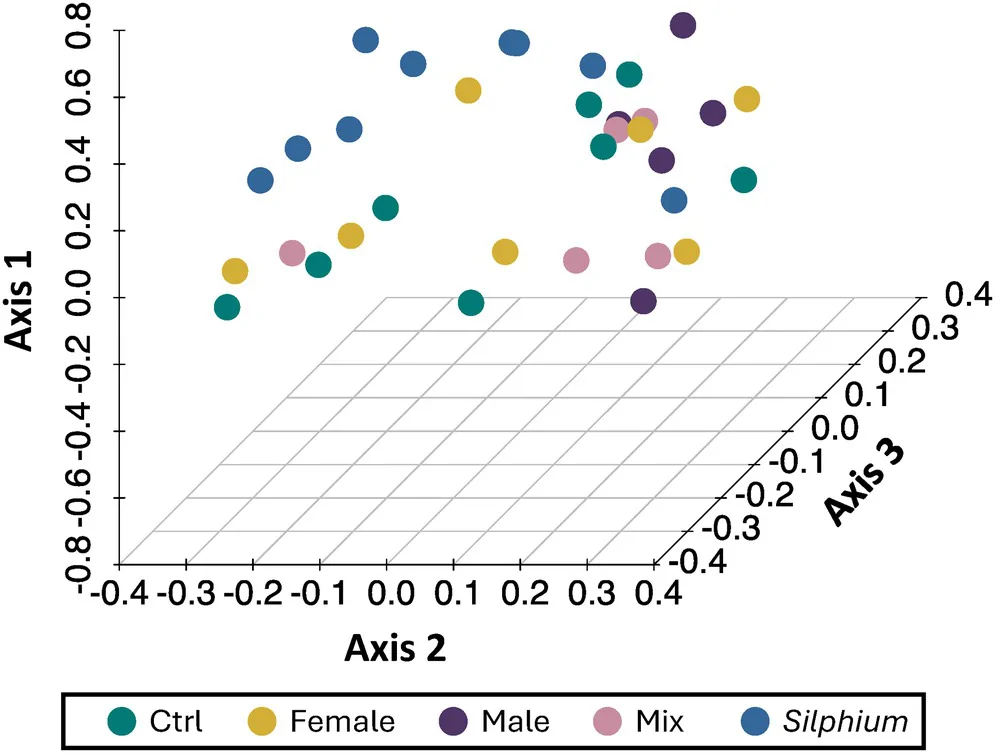
Headspace collected visualized through nonmetric dimensional scaling (NMDS) based on a pairwise distance matrix calculated from a Bray–Curtis index for samples from *Eucosma giganteana* conspecifics (male, female, mix), flower heads of *Silphium integrifolium* , or a blank control. There were a total of *n* = 5–9 replicates per treatment, and stress < 0.13.

**TABLE 4:** Tentatively identified max‐min scaled relative peak areas from cohorts of field‐collected adult *Eucosma giganteana* moths and *Silphium integrifolium* flower heads over a 24 h period in the laboratory.

Compound^a^	RT^b^	Control		Female		Male		Mixed		*S. integrifolium*	
		Mean ± SE	%Total	Mean ± SE	%Total	Mean ± SE	%Total	Mean ± SE	%Total	Mean ± SE	%Total
2‐Methyl‐5,5‐diphenyl‐4‐(methylthio)imidazole	26.0	0.0035 ± 0.0035	1.42	0.0064 ± 0.0044	5.71	0.0039 ± 0.0039	34.2	—	—	—	—
1,3‐Diphenyl‐4H‐1,2,4‐triazoline‐5‐thione	25.5	0.008 ± 0.0008	0.33	0.0015 ± 0.001	1.34	—	—	—	—	—	—
L‐Valine, N‐(2‐furoyl)‐, undecyl ester	25.1	0.1667 ± 0.1667	67.5	0.0138 ± 0.009	12.3	—	—	0.0048 ± 0.0041	9.53	0.0048 ± 0.0042	1.03
N‐Fluoroiminomalonic nitrile	22.1	0.0008 ± 0.0008	0.33	—	—	0.0002 ± 0.0002	1.82	0.0002 ± 0.0002	0.38	0.0001 ± 0.0001	0.02
2,2‐Dimethyl‐propyl 2,2‐dimethyl‐propanesulfinyl sulfone	8.45	0.0001 ± 0.0001	0.04	0.005 ± 0.004	0.42	0.0029 ± 0.0027	25.9	—	—	—	—
1H‐1,2,3‐Triazole‐4‐carboxaldehyde	21.0	—	—	0.0007 ± 0.0005	0.67	—	—	—	—	—	—
Bornyl acetate	9.27	0.0027 ± 0.0015	1.11	—	—	0.0032 ± 0.002	28.5	—	—	0.0005 ± 0.0005	0.1
Carbonic chloride fluoride	8.01	—	—	0.0109 ± 0.0102	9.71	0.0003 ± 0.0003	2.12	0.0013 ± 0.0006	2.52	0.0002 ± 0.0002	0.05
**beta‐Phellandrene** *	**4.91**	—	—	—	—	—	—	**0.0027 ± 0.0027**	**5.37**	**0.0356 ± 0.0119**	**7.58**
**Camphene** *	**4.63**	—	—	—	—	—	—	**0.0170 ± 0.0027**	**34.1**	**0.1997 ± 0.0762**	**42.6**
**D‐Limonene** *	**5.64**	**0.0003 ± 0.0002**	**0.12**	**0.0008 ± 0.0005**	**0.74**	**0.0004 ± 0.0004**	**3.46**	**0.0176 ± 0.0121**	**35.3**	**0.0186 ± 0.0078**	**3.97**
**β‐Pinene** *	**4.98**	**0.0727 ± 0.0390**	**29.4**	**0.0772 ± 0.0488**	**69.0**	—	—	—	—	**0.1114 ± 0.0656**	**23.7**
β‐Thujene		—	—	—	—	—	—	—	—	0.0112 ± 0.0055	2.38
**β‐Myrcene** *	**5.08**	—	—	—	—	—	—	**0.0026 ± 0.0017**	**5.21**	**0.003 ± 0.0017**	**0.63**
1,3‐Butadiyne, 1,4‐difluoro—		—	—	—	—	—	—	0.0031 ± 0.0031	6.28	0.0629 ± 0.0354	12.4
Succinic acid, 2,2,3,3,4,4,4‐heptafluorobutyl 2‐methylhex‐3‐yl ester		—	—	—	—	—	—	—	—	0.0079 ± 0.0029	1.68
1H‐Imidazole, 5‐iodo‐2‐methyl‐4‐nitro—		—	—	—	—	—	—	0.0002 ± 0.0002	0.32	0.0001 ± 0.0001	0.03
Benzenamine, 2‐iodo—		—	—	—	—	0.0004 ± 0.0004	3.86	0.0005 ± 0.0005	1.01	0.0003 ± 0.0003	0.07
**Total scaled peak areas**		0.0309 ± 0.0265	100	0.0124 ± 0.0083	100	0.0016 ± 0.0014	100	0.005 ± 0.0037	100	0.0293 ± 0.0135	100

*Note:* Confirmed compounds are in bolded rows.

^a^ Tentatively identified compounds compared to the NIST14 library.

^b^ Retention time on HP‐5 column.

* Confirmed with commercial standards purchased from Sigma‐Aldrich have an asterisk and are bolded.

### 3.4 *E. giganteana* Electroantennography

There was a significant difference in the antennal depolarization due to the semiochemical treatment for both the male and the female *E. giganteana* (Table 5). The puff sequence did not significantly impact the behavioral response of *E. giganteana* (Table 5, Figure 7). Female *E. giganteana* generally showed greater responsiveness to *S. integrifolium* headspace by 1.8‐fold than to DCM and DCM + tetradecane controls and was generally higher than male antennal response to *S. integrifolium* . There was a 1.5‐fold greater response by female *E. giganteana* to neat (*E*)‐8‐dodecenyl acetate compared to acetone, which was the positive control (Figure 7). Female and male *E. giganteana* exhibited a 0.7‐ to 2.3‐fold and 2.8‐ to 4.1‐fold greater response to (*E*)‐8‐dodecenyl acetate compared to DCM and DCM + tetradecane controls. Moreover, response by female and male *E. giganteana* to neat (*E*)‐8‐dodecenyl acetate was 3.2‐ and 2‐fold higher than the acetone positive control. Puff sequence did not affect overall qualitative patterns of response by females or males (Figure S3). For male moths, there was a 9‐fold difference in response between acetone control and *S. integrifolium* , while there was an 18‐fold change in responsiveness between the acetone control and the opposite sex. The presence of the opposite sex did not significantly elicit antennal response.

**TABLE 5:** Summary of linear mixed model results for *Eucosma giganteana* corrected antennal depolarization for females (*n* = 15) and males (*n* = 17) via EAG semiochemical treatment, stimulus puff, sex, and their two‐way and three‐way interactions.

Factor	df	*F*	*p*
Treatment	4	29.5	0.0001
Stimulus puff	1	1.43	0.231
Sex	1	23.0	0.0001
Treatment × Stimulus puff	4	1.06	0.900
Treatment × Sex	4	71.4	0.0001
Stimulus puff × Sex	1	5.62	0.02
3‐way interaction	4	4.67	0.323

**FIGURE 7:**
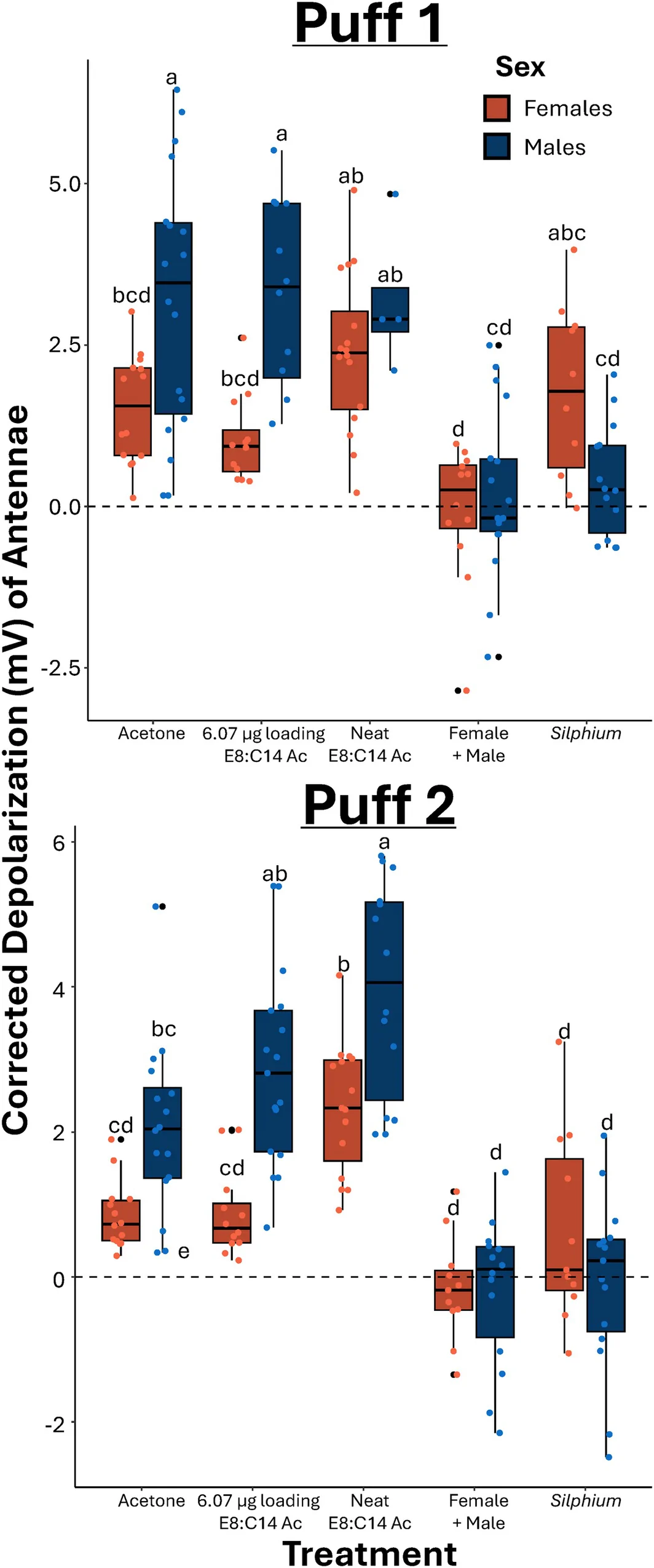
Female (orange) (*n* = 15 biological replicates per treatment) and male (blue) (*n* = 17 biological replicates per treatment) *Eucosma giganteana* corrected antennal depolarization via EAG across the five semiochemical treatments on the first stimulus puff (top panel) and second stimulus puff (bottom panel). Thick black horizontal bar represents median on each boxplot. Bars sharing the same letters are not significantly different among the treatment in the same stimulus puff (Tukey HSD, *α* = 0.05).

### 3.5 Flight Mill

There were no significant differences by date in the average distance flown for either sex of *E. giganteana* nor in the semiochemical treatment to which the individual moths were exposed (Table 6). Similarly, there was no significant difference in the average velocity of flight for either sex of *E. giganteana* across the control and semiochemical treatments (Figure 8). With both sexes, we saw no difference in the average distance flown when the moths were in the presence of the opposite sex (Figure 9). There was no effect of the presence of the opposite sex on distance moved (*F* = 2.69; df = 1, 40; *p* = 0.11) or on the velocity by *E. giganteana* (*F* = 1.74; df = 1, 40; *p* = 0.20; Figure 9). However, male *E. giganteana* that weighed more flew significantly longer distances (Adj. *R* ^2^ = 0.261; *F* = 16.5; df = 43; *p* = 0.0002; Figure 10). Female moths did not share the same correlation between weight and flight distance (Adj. *R* ^2^ = 0.019; *F* = 2.07; df = 53; *p* = 0.16).

**TABLE 6:** Summary of ANOVA results on *Eucosma giganteana* flight capacity by sex, treatment, and date, weight, and the 2‐way interaction of sex by treatment.

Factor	df^a^	*F*	*p*
Sex	1	3.31	0.07
Treatment	3	2.19	0.1
Date	7	1.88	0.09
Weight	1	9.60	0.003
Sex × Treatment	3	1.53	0.21

^a^ Residual df = 92.

**FIGURE 8:**
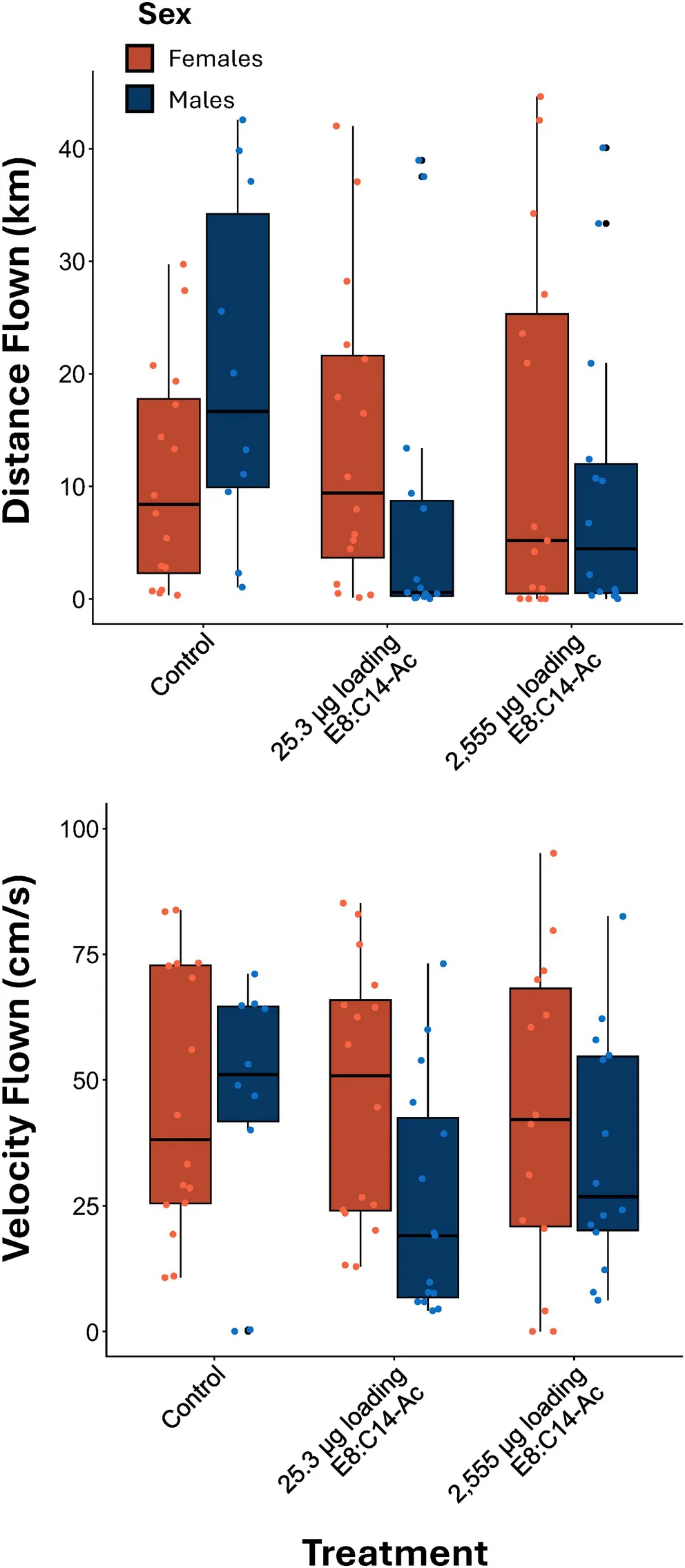
Field‐collected female (orange) and male (blue) *Eucosma giganteana* distance flown (top panel) and velocity (cm/s) in a 24 h period in response to different semiochemical treatments, including 25.3 μg and 2555 μg loadings of (*E*)‐8‐dodecenyl acetate on an automated flight mill in the laboratory under constant conditions (27.5°C, 65% RH, and 16:8 L:D). There were no significant differences among the treatments (*n* = 16 biological replicates per sex and treatment combination).

**FIGURE 9:**
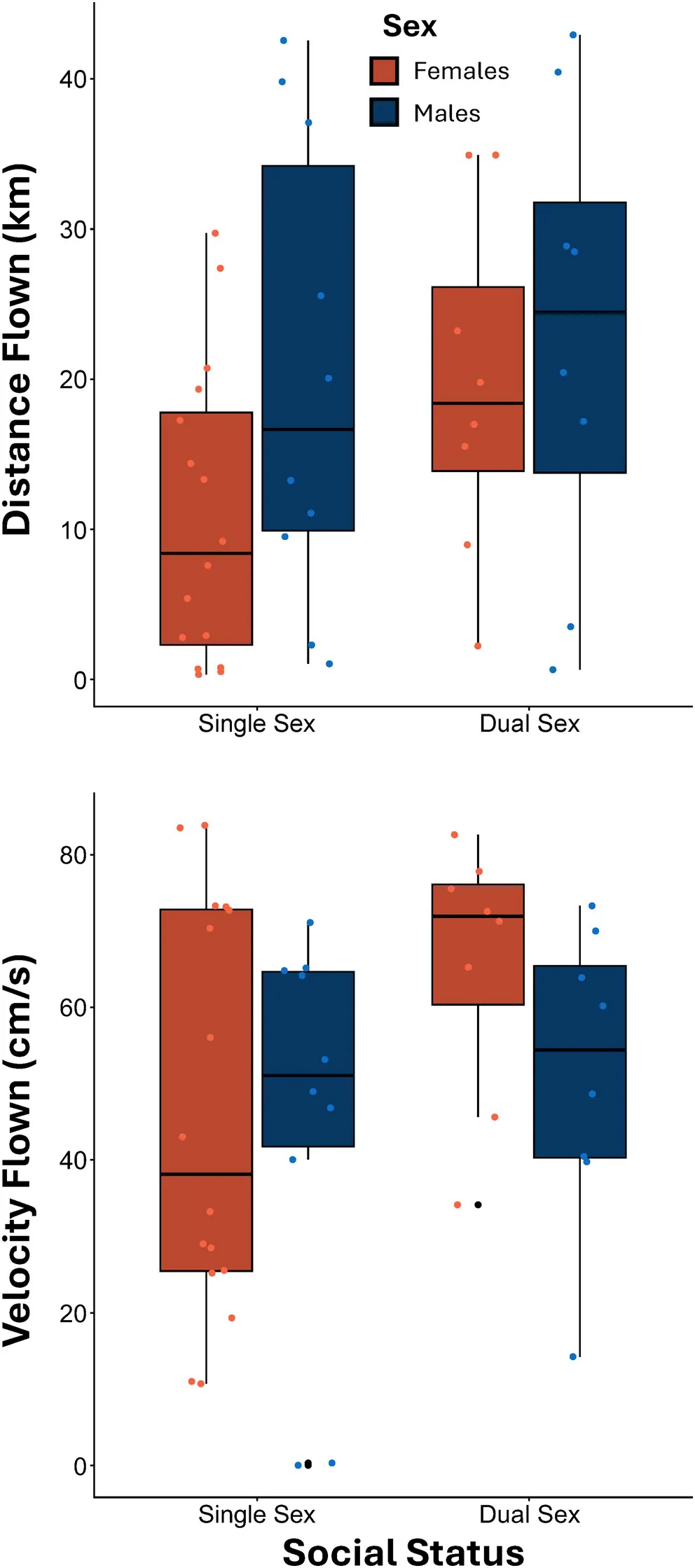
Field‐collected female (orange) and male (blue) *Eucosma giganteana* distance flown (top panel) and velocity (cm/s) in a 24 h period in response to presence with members of own sex only (single sex) or both sexes (dual sex) when no other semiochemicals are present on an automated flight mill in the laboratory under constant conditions (27.5°C, 65% RH, and 16:8 L:D). There were no significant differences among the treatments (*n* = 8 biological replicates per sex). Letters left off of panels with no significant differences among treatments.

**FIGURE 10:**
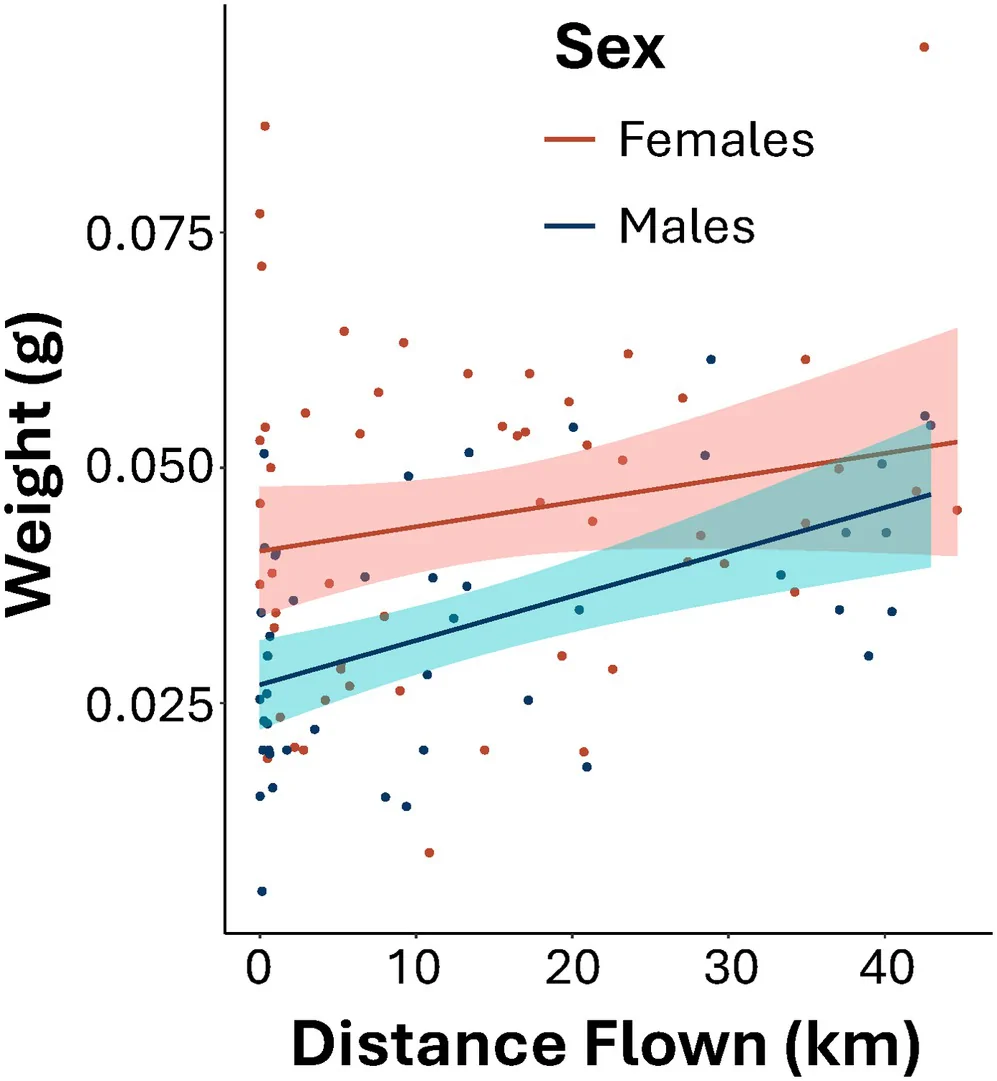
Field‐collected female and male *Eucosma giganteana* showing a correlation between distance flown in a 24 h period and weight (g) on an automated flight mill in the laboratory (*n* = 55 female and 45 males).

## 4 Discussion

This is the first study to evaluate different loadings of (*E*)‐8‐dodecenyl acetate in the field on captures of *E. giganteana* and nontarget Lepidoptera, which has major implications for future research on this pest. We have demonstrated that with increasing loading of (*E*)‐8‐dodecenyl acetate in lures, there are higher captures of *E. giganteana*, but not nontarget Lepidoptera found in Kansas *S. integrifolium* fields. This is an important first step in demonstrating the potential promise of this compound in behaviorally‐based management programs for this pest (e.g., Morrison III et al. 2016), such as attract‐and‐kill or mating disruption (Morrison III et al. 2021). Semiochemical‐mediated behaviorally‐based strategies typically function best when used on intermediate or small‐sized populations of pests, which is what we have observed in the field for *E. giganteana*. Prior work found that among multiple compounds identified from related species in the genus *Eucosma*, only (*E*)‐8‐dodecenyl acetate was found to elicit a response in *E. giganteana* (Ruiz et al. 2022). It is possible that the actual pheromone for this species is structurally similar to (*E*)‐8‐dodecenyl acetate. Further work should be done to determine additional compounds that are attractive to *E. giganteana* to create a more complete pheromone blend.

A low loading of (*E*)‐8‐dodecenyl acetate should be suitable for monitoring *E. giganteana* with clear sticky cards, but its efficacy may decrease toward the end of the season. In addition, when higher loadings of (*E*)‐8‐dodecenyl acetate are used in the same field, it is possible that a low loading may not be sufficient to track populations of *E. giganteana*, as we observed in the field in 2024. While we did not specifically assess whether high loadings of (*E*)‐8‐dodecenyl acetate can prevent males from locating females, the use of this compound for mating disruption could be explored in future studies. Mating disruption using synthetic pheromone blends has been highly effective in managing other tortricid moths in diverse agricultural systems (Amarasekare and Shearer 2017; Bohnenblust et al. 2011; Flores et al. 2021; Knight et al. 2017).

Importantly, we did not see a significant difference between nontarget lepidopteran captures between the control and any of the baited treatments in either year in Kansas fields. This further confirms the specificity of this attractant to *E. giganteana*. It should be noted that (*E*)‐8‐dodecenyl acetate is a component in other tortricid sex pheromone blends including for the plume fruit moth (*Grapholita funebrana* Treitschke) and the oriental fruit moth (*Grapholita molesta* (Busck)) (Lo Verde et al. 2020; Tollerup et al. 2012; Trimble et al. 2001). While this compound could hypothetically lead to more nontarget captures in areas with higher populations of these species in other areas, we did not observe an increase in lepidopteran captures in our study using *S. integrifolium* fields in Kansas.

Changes in the commercial formulation of the sticky cards used in 2023 led to the decline in trap captures around July 26, resulting in an unusual bivoltine pattern of captures instead of the usual univoltine pattern (Ruiz et al. 2022). The dip in captures around July 29th during the 2024 season, could be explained by a heat wave that the area experienced. The 2024 trends were mirrored by the UV light captures conducted for ongoing *E. giganteana* monitoring (N. Chérémond personal communication), indicating it was not specific to the sticky cards. In the future, it may be useful to consider multiple modalities (e.g., light and pheromone) to trap *E. giganteana*, as some species have shown increased sensitivity to traps with more than one modality. For example, adding an LED light to pheromone‐baited traps increased tortricid moth captures by 2‐ to 12‐fold in pome fruit (Knight et al. 2023). Follow‐up experimentation should be conducted examining the relationship between trap captures with UV light and the attractants used in this study.

During this study, we have demonstrated that tentatively identified volatile bouquets from *E. giganteana* and its host crop are significantly different. In addition, we have determined that *E. giganteana* females exhibited enhanced antennal responsiveness to *S. integrifolium* , while males do not. Both sexes show elevated antennal response to (*E*)‐8‐dodecenyl acetate through EAG. However, there was some variability in response from the individual antennae tested to the different stimuli, so more work should be done to verify these results. While the EAG can confirm that a species perceives a volatile blend, it is limited in having any bearing on the behavioral response (Brezolin et al. 2018; Lu et al. 2024). However, we have shown in the present study that (*E*)‐8‐dodecenyl acetate is attractive to *E. giganteana* in the field, which is also confirmed by prior literature (Ruiz et al. 2022). Our results showed that both sexes do appear to have a strong response to (*E*)‐8‐dodecenyl acetate, which suggests that both sexes perceive this compound. Future work should be done examining if female *E. giganteana* alter their pheromone calling strategies when in the presence of other female *E. giganteana* or (*E*)‐8‐dodecenyl acetate (Chen et al. 2014; Knight et al. 2017; Witzgall et al. 2010).

The flight mill showed *E. giganteana* response followed a similar pattern of flight in terms of total distance flown as those in Ruiz et al. (2022). However, Ruiz et al. (2022) also found evidence of arrestment behavior in *E. giganteana* on the flight mill in the presence of (*E*)‐8‐dodecenyl acetate and showed a greater arrestment effect for male moths. It was interesting to note that there was no significant difference in flight activity between recording dates, indicating that the moths collected at the beginning and end of the flight period were equally active on the flight mill in the laboratory under the tested conditions. Although some other species of insects require dispersal periods early in the season (e.g., *Halyomorpha halys* ; Bergh et al. 2017), it is unknown whether *E. giganteana* requires one. However, this possibility should be specifically tested in the future. The variability of using field caught specimens could have contributed to the lack of significance and large standard errors, including variation in age and mating status of moths since their history was unknown. Previous studies on other tortricid species have found that age and mating status influence the moth's distance flown and propensity to fly (Schumacher et al. 1997; Su et al. 2022). In the future, moths reared from pupae should be tested, so that flight capacity in response to age and mating status can be evaluated.

We found *S. integrifolium* scaled relative peak areas were strongly separated from the other headspace treatments. Overall, *S. integrifolium* headspace was much more complex and contained a wider variety in classes of compounds. It primarily consisted of 10 compounds that were relatively unique, with those in highest abundance confirmed as camphene and beta‐phellandrene, and possibly 1,4‐difluoro‐1,3‐butadiyne, which was tentatively identified, but not confirmed. Interestingly, the confirmed camphene was also enriched in the headspace of mixed‐sex *E. giganteana*, and we hypothesize that moths may produce or sequester camphene and use it in intraspecific communication, but this needs to be explicitly tested in future years. Overall, the lack of differences seen among the other semiochemical treatments may be the result of using a density of *E. giganteana* that was too low in headspace collection jars. A higher density may be required, as some experiments require 40–100 moth individuals (Stanley et al. 2018; Zagrobelny et al. 2015). A higher density of individuals could also have increased the antennal response of the headspace during the EAG experiment. Future studies should also perform microdissections of the sex pheromone gland, likely located between the eighth and ninth abdominal segments. Overall, the field experiments support (*E*)‐8‐dodecenyl acetate as a candidate monitoring lure, with higher nominal loadings producing greater captures in the 2024 experiment. EAG responses provide exploratory evidence of antennal detection under the fixed presentation sequence, whereas the flight mill assay detected no treatment effect under the tested conditions. Additional behavioral work is needed before drawing conclusions about sex‐specific attraction, autodetection, mating disruption, or attract‐and‐kill. These results help us better understand how *E. giganteana* reacts to different semiochemical stimuli and lays a foundation for the development of behaviorally‐based management tactics.

## Author Contributions

**Hazel Scribner:** conceptualization (equal), data curation (lead), formal analysis (lead), investigation (lead), methodology (equal), visualization (equal), writing – original draft (lead), writing – review and editing (lead). **Jacqueline Maille:** formal analysis (supporting), methodology (equal), resources (supporting), software (lead), writing – review and editing (equal). **Ebony G. Murrell:** conceptualization (equal), methodology (supporting), project administration (supporting), resources (lead), supervision (supporting), writing – review and editing (equal). **Matthew Hetherington:** formal analysis (supporting), investigation (supporting), methodology (equal), software (supporting). **Erin D. Scully:** methodology (supporting), project administration (supporting), resources (supporting), writing – review and editing (equal). **Kun Yan Zhu:** funding acquisition (equal), project administration (equal), resources (equal), supervision (equal), writing – review and editing (equal). **William R. Morrison III:** conceptualization (lead), formal analysis (supporting), funding acquisition (lead), project administration (lead), resources (lead), supervision (lead), visualization (supporting), writing – review and editing (equal).

## Funding

This work was funded by the Perennial Agriculture Project (PAP) from the Malone Foundation and governed by a NCFA between KSU and USDA‐ARS (agreement no. 58‐3020‐2‐004‐N).

## Conflicts of Interest

The authors declare no conflicts of interest.

## Supporting information


**Table S1:** Experimental design and number of treatments for 2023 and 2024 baiting field trials in Salina, KS with clear yellow sticky cards for *Eucosma giganteana*.
**Table S2:** Summary of stock solutions, concentrations, application volumes, and loading rates for each experiment testing behavioral and physiological response by *E. giganteana*.
**Table S3:** Pairwise assessment of independence of trap loctions in 2023 during a field trapping experiment for *E. giganteana* in *Silphium integrifolium* fields in Salina, KS using Spearman ρ correlations. Warmer colors (=red) indicate lower *p*‐values, while cooler colors (=blue) indicate higher *p*‐values.
**Table S4:** Pairwise assessment of independence of trap loctions in 2024 during a field trapping experiment for *E. giganteana* in *Silphium integrifolium* fields in Salina, KS using Spearman ρ correlations. Warmer colors (=red) indicate lower *p*‐values, while cooler colors (=blue) indicate higher *p*‐values.
**Figure S1:** Comparison of *E. giganteana* moth weights among semiochemical treatments in the flight mill assay. There was no significant difference in weights of moths among any of the treatments tested.
**Figure S2:** Representative mass spectra showing the *m*/*z* ratio against abundance from isolated *Silphium* samples (top row), a commercially purchased standard from Supelco (Terpene Mix A; middle row), and the relative abundance from NIST14 reference hits (bottom row). The compounds β‐myrcene, β‐phellandrene, β‐pinene, camphene, and limonene were confirmed.
**Figure S3:** Corrected antennal depolarization for the first (darker) and second (puff) of each treatment by female (orange) (*n* = 15 biological replicates per treatment) and male (blue) (*n* = 17 biological replicates per treatment) *E. giganteana* via EAG across the seven semiochemical treatments. Thick black horizontal bar represents median on each boxplot. Couplet boxplots with “ns” are not significantly different from each other (Tukey HSD, *α* = 0.05).

**Data S1:** Supporting Information

## Data Availability

All the data in this study were deposited per USDA regulations in AgData Commons as follows: Scribner et al. (2024), https://doi.org/10.15482/USDA.ADC/28055111.v1.

## References

[ece374476-bib-0001] Abshire, J. , R. Harman , A. Bruce , et al. 2024. “Flight Capacity and Behavior of *Ephestia kuehniella* (Lepidoptera: Pyralidae) in Response to Kairomonal and Pheromonal Stimuli.” Environmental Entomology 53, no. 4: 567–576. 10.1093/ee/nvae039. 38704359

[ece374476-bib-0002] Amarasekare, K. , and P. Shearer . 2017. “Stability of *Cacopsylla pyricola* (Hemiptera: Psyllidae) Populations in Pacific Northwest Pear Orchards Managed With Long‐Term Mating Disruption for *Cydia pomonella* (Lepidoptera: Tortricidae).” Insects 8, no. 4: 105. 10.3390/insects8040105. PMC5746788 28974000

[ece374476-bib-0003] Attisano, A. , J. T. Murphy , A. Vickers , and P. J. Moore . 2015. “A Simple Flight Mill for the Study of Tethered Flight in Insects.” Journal of Visualized Experiments 106: 53377. 10.3791/53377. PMC4692786 26709537

[ece374476-bib-0004] Bates, D. , M. Mächler , B. Bolker , and S. Walker . 2015. “Fitting Linear Mixed‐Effects Models Using lme4.” Journal of Statistical Software 67, no. 1: 1–48. 10.18637/jss.v067.i01.

[ece374476-bib-0005] Bergh, J. C. , W. R. Morrison III , S. V. Joseph , and T. C. Leskey . 2017. “Characterizing Spring Emergence of Adult *Halyomorpha halys* Using Experimental Overwintering Shelters and Commercial Pheromone Traps.” Entomologia Experimentalis et Applicata 162, no. 3: 336–345.

[ece374476-bib-0006] Bohnenblust, E. , L. A. Hull , and G. Krawczyk . 2011. “A Comparison of Various Mating Disruption Technologies for Control of Two Internally Feeding Lepidoptera in Apples: Mating Disruption of Two Moth Species.” Entomologia Experimentalis et Applicata 138, no. 3: 202–211. 10.1111/j.1570-7458.2010.01089.x.

[ece374476-bib-0007] Brezolin, A. N. , J. Martinazzo , D. K. Muenchen , et al. 2018. “Tools for Detecting Insect Semiochemicals: A Review.” Analytical and Bioanalytical Chemistry 410, no. 17: 4091–4108. 10.1007/s00216-018-1118-3. 29926152

[ece374476-bib-0008] Casado, D. , C. Gemeno , J. Avilla , and M. Riba . 2006. “Day‐Night and Phenological Variation of Apple Tree Volatiles and Electroantennogram Responses in *Cydia pomonella* (Lepidoptera: Tortricidae).” Environmental Entomology 35, no. 2: 258–267. 10.1603/0046-225X-35.2.258.

[ece374476-bib-0009] Chen, R.‐Z. , M. G. Klein , C.‐F. Sheng , et al. 2014. “Mating Disruption or Mass Trapping, Compared With Chemical Insecticides, for Suppression of *Chilo suppressalis* (Lepidoptera: Crambidae) in Northeastern China.” Journal of Economic Entomology 107, no. 5: 1828–1838. 10.1603/EC14148. 26309273

[ece374476-bib-0010] Flores, M. F. , J. Bergmann , C. Ballesteros , D. Arraztio , and T. Curkovic . 2021. “Development of Monitoring and Mating Disruption Against the Chilean Leafroller *Proeulia auraria* (Lepidoptera: Tortricidae) in Orchards.” Insects 12, no. 7: 625. 10.3390/insects12070625. PMC8307431 34357285

[ece374476-bib-0011] Frerot, B. , E. Priesner , and M. Gallois . 1979. “A Sex Attractant for the Green Budworm Moth, Hedya Nubiferana.” Zeitschrift fur Naturforschung C: Journal of Biosciences 34, no. 12: 1248–1252. 10.1515/znc-1979-1229.

[ece374476-bib-0012] Fu, N. , F. H. Magsi , Y. Zhao , et al. 2022. “Identification and Field Evaluation of Sex Pheromone Components and Its Antagonist Produced by a Major Tea Pest, *Archips strojny* (Lepidoptera: Tortricidae).” Insects 13, no. 11: 1056. 10.3390/insects13111056. PMC9697133 36421959

[ece374476-bib-0013] Fyie, J. Q. , C. A. Stratton , W. R. Morrison III , and E. G. Murrell . 2025. “Intercropping Alters Phytochemical Defenses Against Insect Herbivory.” Journal of Chemical Ecology 51, no. 2: 46. 10.1007/s10886-025-01555-9. 40164956

[ece374476-bib-0017] Hughes, J. , A. Hern , and S. Dorn . 2004. “Preimaginal environment influences adult flight in *Cydia molesta* (Lepidoptera: Tortricidae).” Environmental Entomology 33, no. 5: 1155–1162. 10.1603/0046-225X-33.5.1155.

[ece374476-bib-0018] Hung, C. C. , J. S. Hwang , M. D. Hung , Y. P. Yen , and R. F. Hou . 2001. “Isolation, Identification and Field Tests of the Sex Pheromone of the Carambola Fruit Borer, *Eucosma notanthes* .” Journal of Chemical Ecology 27, no. 9: 1855–1866. 10.1023/A:1010464810443. 11545375

[ece374476-bib-0019] Johnson, P. J. , A. Boe , and A. P. Martens . 2019. “ *Eucosma giganteana* (Riley) and *Sliphium perfoliatum* L., Morphological Variation in an Insect‐Plant Association in Eastern South Dakota.” Proceedings of the South Dakota Academy of Science 98: 91–104.

[ece374476-bib-0020] Jones, H. B. C. , K. S. Lim , J. R. Bell , J. K. Hill , and J. W. Chapman . 2016. “Quantifying Interspecific Variation in Dispersal Ability of Noctuid Moths Using an Advanced Tethered Flight Technique.” Ecology and Evolution 6, no. 1: 181–190. 10.1002/ece3.1861. PMC4716516 26811783

[ece374476-bib-0021] Knight, A. L. , A. M. El‐Sayed , G. J. R. Judd , and E. Basoalto . 2017. “Development of 2‐Phenylethanol Plus Acetic Acid Lures to Monitor Obliquebanded Leafroller (Lepidoptera: Tortricidae) Under Mating Disruption.” Journal of Applied Entomology 141, no. 9: 729–739. 10.1111/jen.12393.

[ece374476-bib-0022] Knight, A. L. , R. Hilton , and D. M. Light . 2005. “Monitoring Codling Moth (Lepidoptera: Tortricidae) in Apple With Blends of Ethyl (E,Z)‐2, 4‐Decadienoate and Codlemone.” Environmental Entomology 34: 598–603.

[ece374476-bib-0023] Knight, A. L. , M. Preti , E. Basoalto , and E. Fuentes‐Contreras . 2023. “Increasing Catches of Adult Moth Pests (Lepidoptera: Tortricidae) in Pome Fruit With Low‐Intensity led Lights Added to Sex Pheromone/Kairomone Lure‐Baited Traps.” Journal of Applied Entomology 147, no. 9: 843–856. 10.1111/jen.13176.

[ece374476-bib-0025] Lenth, R. , and J. Piaskowski . 2026. “emmeans: Estimated Marginal Means, aka Least‐Squares Means.” R package version 2.0.5. https://rvlenth.github.io/emmeans/.

[ece374476-bib-0026] Lo Verde, G. , S. Guarino , S. Barone , and R. Rizzo . 2020. “Can Mating Disruption Be a Possible Route to Control Plum Fruit Moth in Mediterranean Environments?” Insects 11, no. 9: 589. 10.3390/insects11090589. PMC7564571 32882909

[ece374476-bib-0027] Lu, H. , Q. Wu , Y.‐Q. Yang , et al. 2024. “(*Z*, *Z*, *Z*)‐3,6,9‐Nonadecadiene, a Potential Inhibitor of Sex Pheromone of Grey Tea Geometrid (Lepidoptera: Geometridae): Electroantennogram Test, Wind Tunnel, and in Silico Study.” Journal of Economic Entomology 117, no. 3: 793–799. 10.1093/jee/toae033. 38517276

[ece374476-bib-0028] Matveev, E. , J. J. Kwon , G. J. R. Judd , and M. L. Evenden . 2017. “The Effect of Cold Storage of Mass‐Reared Codling Moths (Lepidoptera: Tortricidae) on Subsequent Flight Capacity.” Canadian Entomologist 149, no. 3: 391–398. 10.4039/tce.2017.1.

[ece374476-bib-0029] McNair, C. , G. Gries , and R. Gries . 1999. “Sex Pheromone Components of *Enarmonia formosana* (Lepidoptera: Tortricidae).” Canadian Entomologist 131, no. 1: 85–92. 10.4039/Ent13185-1.

[ece374476-bib-0030] Miller, J. R. , C. G. Adams , P. A. Weston , and J. H. Schenker . 2015. Trapping of Small Organisms Moving Randomly: Principles and Applications to Pest Monitoring and Management. Springer International Publishing (SpringerBriefs in Ecology). 10.1007/978-3-319-12994-5.

[ece374476-bib-0031] Morrison, W. R., III , D.‐H. Lee , B. D. Short , A. Khrimian , and T. C. Leskey . 2016. “Establishing the Behavioral Basis for an Attract‐And‐Kill Strategy to Manage the Invasive *Halyomorpha halys* in Apple Orchards.” Journal of Pest Science 89, no. 1: 81–96. 10.1007/s10340-015-0679-6.

[ece374476-bib-0032] Morrison, W. R., III , E. D. Scully , and J. F. Campbell . 2021. “Towards Developing Areawide Semiochemical‐Mediated, Behaviorally‐Based Integrated Pest Management Programs for Stored Product Insects.” Pest Management Science 77, no. 6: 2667–2682. 10.1002/ps.6289. 33481331

[ece374476-bib-0033] Murrell, E. G. , K. R. Zio , N. E. Chérémond , and D. L. Van Tassel . 2023. “Assessing Effective Mechanical and Chemical Strategies for Managing *Eucosma giganteana* (Lepidoptera: Tortricidae) in the Perennial Oilseed Crop, *Silphium integrifolium* (Asteraceae: Heliantheae).” Journal of Insect Science 23, no. 6: 4. 10.1093/jisesa/iead102. PMC10661661 37987629

[ece374476-bib-0034] Naranjo, S. E. 2019. “Assessing Insect Flight Behavior in the Laboratory: A Primer on Flight Mill Methodology and What Can Be Learned.” Annals of the Entomological Society of America 112, no. 3: 182–199. 10.1093/aesa/say041.

[ece374476-bib-0035] Oksanen, J. , G. Simpson , F. Blanchet , et al. 2024. “vegan: Community Ecology Package.” https://CRAN.R‐project.org/package=vegan.

[ece374476-bib-0036] Prasifka, J. R. , R. E. Mallinger , B. S. Hulke , S. R. Larson , and D. L. Van Tassel . 2017. “Plant–Herbivore and Plant–Pollinator Interactions of the Developing Perennial Oilseed Crop, *Silphium integrifolium* .” Environmental Entomology 46, no. 6: 1339–1345. 10.1093/ee/nvx134. 29029088

[ece374476-bib-0037] Price, J. H. , D. L. Van Tassel , V. D. Picasso , and K. P. Smith . 2022. “Assessing Phenotypic Diversity in Silflower ( *Silphium integrifolium* Michx.) to Identify Traits of Interest for Domestication Selection.” Crop Science 62, no. 4: 1443–1460. 10.1002/csc2.20748.

[ece374476-bib-0038] Reinert, S. , D. L. Van Tassel , B. Schlautman , N. C. Kane , and B. S. Hulke . 2019. “Assessment of the Biogeographical Variation of Seed Size and Seed Oil Traits in Wild *Silphium integrifolium* Michx. Genotypes.” Plant Genetic Resources 17, no. 5: 427–436. 10.1017/S1479262119000248.

[ece374476-bib-0039] Ruiz, K. , A. Bruce , N. Chérémond , et al. 2022. “Field Trapping and Flight Capacity of *Eucosma giganteana* (Riley) (Lepidoptera: Tortricidae) in Response to Behaviorally Active Congeneric Semiochemicals in Novel Silflower Agroecosystems.” Insects 13, no. 4: 350. 10.3390/insects13040350. PMC9032278 35447794

[ece374476-bib-0040] Schoonhoven, L. M. , J. J. A. van Loon , and M. Dicke . 2005. Insect‐Plant Biology. Oxford University Press.

[ece374476-bib-0041] Schumacher, P. , A. Weyeneth , D. C. Weber , and S. Dorn . 1997. “Long Flights in *Cydia pomonella* L. (Lepidoptera: Tortricidae) Measured by a Flight Mill: Influence of Sex, Mated Status and Age.” Physiological Entomology 22, no. 2: 149–160. 10.1111/j.1365-3032.1997.tb01152.x.

[ece374476-bib-0042] Scribner, H. , J. M. Maille , E. G. Murrell , et al. 2024. “Behavioral and Physiological Response of *Eucosma giganteana* to Semiochemicals From Conspecifics and *S. integrifolium.”* Ag Data Commons. Dataset. 10.15482/USDA.ADC/28055111.v1.

[ece374476-bib-0043] Scribner, H. , E. G. Murrell , N. E. Chérémond , et al. 2025. “Developing a Growing Degree Day Model to Guide Integrated Pest Management of *Eucosma giganteana*, a Pest of a Novel Perennial Oilseed Crop.” Journal of Economic Entomology 118, no. 4: 1711–1720. 10.1093/jee/toaf107. 40577681

[ece374476-bib-0044] Stanley, J. , S. Chandrasekaran , G. Preetha , and K. Subaharan . 2018. “Evidence of Male Pheromone in *Conogethes punctiferalis* (Lepidoptera: Pyralidae).” Journal of Entomological Science 53, no. 4: 455–466. 10.18474/JES17-98.1.

[ece374476-bib-0045] Stowe, H. E. , J. P. Michaud , and T. N. Kim . 2022. “Resource Amount and Discontinuity Influence Flight and Reproduction in *Hippodamia convergens* (Coleoptera: Coccinellidae).” Ecosphere 13, no. 9: e4250.

[ece374476-bib-0046] Stratton, C. A. , Y. Thompson , K. Zio , W. R. Morrison III , and E. G. Murrell . 2024. “uafR: An R Package That Automates Mass Spectrometry Data Processing.” PLoS One 19, no. 7: e0306202. 10.1371/journal.pone.0306202 PMC11226021 38968199

[ece374476-bib-0047] Su, S. , X. Zhang , J. Zhang , et al. 2022. “Flight Performance, Fecundity, and Ovary Development of *Grapholita molesta* (Lepidoptera: Torticidae) at Different Ages.” Insects 13, no. 9: 837. 10.3390/insects13090837. PMC9501558 36135538

[ece374476-bib-0048] Tollerup, K. E. , A. Rucker , and P. W. Shearer . 2012. “Whole‐Farm Mating Disruption to Manage *Grapholita molesta* (Lepidoptera: Tortricidae) in Diversified New Jersey Orchards.” Journal of Economic Entomology 105, no. 5: 1712–1718. 10.1603/EC11419. 23156168

[ece374476-bib-0049] Trimble, R. M. , D. J. Pree , and N. J. Carter . 2001. “Integrated Control of Oriental Fruit Moth (Lepidoptera: Tortricidae) in Peach Orchards Using Insecticide and Mating Disruption.” Journal of Economic Entomology 94, no. 2: 476–485. 10.1603/0022-0493-94.2.476. 11332842

[ece374476-bib-0050] Van Tassel, D. L. , K. A. Albrecht , J. D. Bever , et al. 2017. “Accelerating *Silphium* Domestication: An Opportunity to Develop New Crop Ideotypes and Breeding Strategies Informed by Multiple Disciplines.” Crop Science 57, no. 3: 1274–1284. 10.2135/cropsci2016.10.0834.

[ece374476-bib-0051] Vilela, A. , L. González‐Paleo , K. Turner , et al. 2018. “Progress and Bottlenecks in the Early Domestication of the Perennial Oilseed *Silphium integrifolium* , a Sunflower Substitute.” Sustainability 10, no. 3: 638. 10.3390/su10030638.

[ece374476-bib-0052] Vilela, A. E. , L. González‐Paleo , D. A. Ravetta , E. G. Murrell , and D. L. Van Tassel . 2020. “Balancing Forage Production, Seed Yield, and Pest Management in the Perennial Sunflower *Silphium integrifolium* (Asteraceae).” Agronomy 10, no. 10: 1471. 10.3390/agronomy10101471.

[ece374476-bib-0053] Wickham, H. 2016. Elegant Graphics for Data Analysis. Springer‐Verlag.

[ece374476-bib-0054] Witzgall, P. , P. Kirsch , and A. Cork . 2010. “Sex Pheromones and Their Impact on Pest Management.” Journal of Chemical Ecology 36, no. 1: 80–100. 10.1007/s10886-009-9737-y. 20108027

[ece374476-bib-0055] Zagrobelny, M. , H. T. Simonsen , C. E. Olsen , S. Bak , and B. L. Møller . 2015. “Volatiles From the Burnet Moth *Zygaena filipendulae* (Lepidoptera) and Associated Flowers, and Their Involvement in Mating Communication.” Physiological Entomology 40, no. 4: 284–295. 10.1111/phen.12113.

[ece374476-bib-0056] Zhonghui, G. 2021. “ZhonghuiGai/veganEx.” https://rdrr.io/github/ZhonghuiGai/veganEx/.

