# Supplementary material for: Behavioral and Physiological Response of Eucosma giganteana to Semiochemicals From Conspecifics and Silphium integrifolium
Source: Ecol Evol. 2026 Oct 28;16(11):e74476. doi: 10.1002/ece3.74476 (PMC13714635; doi:10.1002/ece3.74476)
Supplement: Supplementary file 1 [file ECE3-16-e74476-s002.docx]

***Scribner et al. Supplementary Materials and Results***

| **Supplementary Table S1.** Experimental design and number of treatments for 2023 and 2024 baiting field trials in Salina, KS with clear yellow sticky cards for *Eucosma giganteana*. | | | | | | | | | | | | | | | |
| --- | --- | --- | --- | --- | --- | --- | --- | --- | --- | --- | --- | --- | --- | --- | --- |
|  |  |  | Per Week^1^ | | | | | |  | Over the Season^2,3^ | | | | | |
| **Year** | **Field** | **No. Transects** | **No. Ctrl Traps** | **No. Low-Loaded Traps per Transect** | **No. Double-Loaded Traps per Transect** | **No. Medium-Loaded Traps**^4^ | **No. High-loaded Traps** | **Weekly Total Traps** |  | **No. Ctrl Traps** | **No. Low-Loaded Traps per Transect** | **No. Double-Loaded Traps per Transect** | **No. Medium-Loaded Traps** | **No. High-loaded Traps** | **Season-Long Total Traps** |
| 2023 | A | 2 | 6 | 4 | 4 | - | - | 14 |  | 45 | 40 | 36 | - | - | 121 |
| 2023 | B1 | 2 | 6 | 4 | 4 | - | - | 14 |  | 45 | 38 | 38 | - | - | 121 |
| 2023 | B2 | 2 | 6 | 4 | 4 | - | - | 14 |  | 47 | 36 | 38 | - | - | 121 |
| 2024 | B3 | 3 | 3 | 3 | - | 3 | 3 | 12 |  | 27 | 27 | - | 27 | 27 | 108 |
| 2024 | C | 3 | 3 | 3 | - | 3 | 3 | 12 |  | 25 | 25 | - | 25 | 25 | 100 |
| 2024 | D | 3 | 3 | 3 | - | 3 | 3 | 12 |  | 27 | 27 | - | 27 | 26 | 107 |
| ^1^ The first 2 weeks in 2023 (6/16 and 6/28) were only control traps without (*E*)-8-dodecenyl acetate, because it was technically prior to the first recorded capture of *E. giganteana* for the season. Subsequently, all treatments were deployed as listed in the table based on first recorded capture of *E. giganteana* in a UV light trap located on the same farm.  ^2^ Numbers over season may not align perfectly based on weekly numbers, because of missing traps as a result of high winds or mowing.  ^3^ Based on sampling for 8–10 weeks during the *E. giganteana* flight period.  ^4^ Hyphen indicates treatment was not deployed in given year. | | | | | | | | | | | | | | |  |

| **Supplementary Table S2.** Summary of stock solutions, concentrations, application volumes, and loading rates for each experiment testing behavioral and physiological response by *E. giganteana*. | | | | |
| --- | --- | --- | --- | --- |
| **Treatment** | **Stock Concentration of E8-C14 Ac. (μl/mL)^1^** | **Total Stock Volume (mL)** | **Application Volume** | **Loaded Mass (μg^1^)** |
| *Field Baiting Experiment*^2^ |  |  |  |  |
| Acetone control | - | - | 50 μL | - |
| Low E8-C14 Ac. | 1.15 | 3 mL | 50 μL | 50.7 |
| Doubled E8-C14 Ac. | 2.3 | 3 mL | 50 μL | 101 |
| Medium E8-C14 Ac. | 15.7 | 3 mL | 50 μL | 692 |
| High E8-C14 Ac. | 116 | 3 mL | 50 μL | 5113 |
| *EAG Experiment*^3^ |  |  |  |  |
| Blank | - | - | - | - |
| DCM + tetradecane control | - | - | 6 μL | - |
| Acetone | - | - | 6 μL | - |
| Low E8-C14 Ac. | 1.15 | 3 mL | 6 μL | 6.07 |
| Neat (high) E8-C14 Ac. | - | - | 6 μL | 5,022 |
| Female + Male headspace | 1 porapak-Q elution | 150 μl | 6 μL | 16.7 ng^4^ or 1.63 ng^5^ |
| Silphium headspace | 1 porapak-Q elution | 150 μl | 6 μL | 65.7 ng |
| *Flight Mill Experiment*^6^ |  |  |  |  |
| Acetone control | - | - | 25 μL | - |
| Low E8-C14 Ac. | 1.15 | 3 mL | 25 μL | 25.3 |
| High E8-C14 Ac. | 116 | 3 mL | 25 μL | 2,555 |
| Female + Male headspace | 4 females and 4 males | 150 μl | intact moths | ~1.12 ng^4^ or 0.11 ng^5,7^ |
| ^1^ Unless otherwise noted |  |  |  |  |
| ^2^ Treatments added to a 3-mL capacity dropping bottle. | | | | |
| ^3^ Treatments added to a 2.5-cm diameter filter paper. | | | | |
| ^4^ Calculated mean emissions of total volatiles in ng over 24 h given the application rate from female *E. giganteana* headspace in GC-MS. | | | | |
| ^5^ Calculated mean emissions of total volatiles in ng over 24 h given the application rate from male *E. giganteana* headspace in GC-MS. | | | | |
| ^6^ Treatments added to a 10 cm diameter filter paper in an open Petri dish. | | | | |
| ^7^ Estimated by dividing the number of moths included in the GC-MS headspace collections to get the per individual emissions and multiplying by four | | | | |

| **Supplementary Table S3.** Pairwise assessment of independence of trap loctions in 2023 during a field trapping experiment for *E. giganteana* in *Silphium integrifolium* fields in Salina, KS using Spearman ρ correlations. Warmer colors (=red) indicate lower P-values, while cooler colors (=blue) indicate higher P-values. | | | |
| --- | --- | --- | --- |
| **Pairwise Comparison** | | *ρ* | *P* |
| Trap Location 1 | Trap Location 2 |  |  |
| A14 | A25 | 1 | 0.0001 |
| A14 | A26 | 1 | 0.0001 |
| A25 | A26 | 1 | 0.0001 |
| A14 | B112 | 1 | 0.0001 |
| A25 | B112 | 1 | 0.0001 |
| A26 | B112 | 1 | 0.0001 |
| A14 | B121 | 1 | 0.0001 |
| A25 | B121 | 1 | 0.0001 |
| A26 | B121 | 1 | 0.0001 |
| B112 | B121 | 1 | 0.0001 |
| A13 | B125 | 1 | 0.0001 |
| A21 | B127 | 1 | 0.0001 |
| A15 | B214 | 1 | 0.0001 |
| B123 | B217 | 1 | 0.0001 |
| B213 | B222 | 1 | 0.0001 |
| A14 | A23 | 0.99 | 0.0017 |
| A23 | A25 | 0.99 | 0.0017 |
| A23 | A26 | 0.99 | 0.0017 |
| A23 | B112 | 0.99 | 0.0017 |
| A23 | B121 | 0.99 | 0.0017 |
| B117 | B225 | 0.96 | 0.1 |
| A11 | A13 | 0.15 | 1 |
| A11 | A14 | 0.75 | 1 |
| A13 | A14 | 0.33 | 1 |
| A11 | A15 | −0.29 | 1 |
| A13 | A15 | −0.22 | 1 |
| A14 | A15 | −0.22 | 1 |
| A11 | A16 | 0.15 | 1 |
| A13 | A16 | −0.33 | 1 |
| A14 | A16 | 0.33 | 1 |
| A15 | A16 | 0.65 | 1 |
| A11 | A17 | −0.065 | 1 |
| A13 | A17 | 0 | 1 |
| A14 | A17 | 0.14 | 1 |
| A15 | A17 | 0.66 | 1 |
| A16 | A17 | 0.87 | 1 |
| A11 | A21 | 0.49 | 1 |
| A13 | A21 | −0.22 | 1 |
| A14 | A21 | 0.65 | 1 |
| A15 | A21 | −0.14 | 1 |
| A16 | A21 | 0.65 | 1 |
| A17 | A21 | 0.47 | 1 |
| A11 | A22 | 0.77 | 1 |
| A13 | A22 | 0.53 | 1 |
| A14 | A22 | 0.79 | 1 |
| A15 | A22 | −0.43 | 1 |
| A16 | A22 | 0 | 1 |
| A17 | A22 | −0.029 | 1 |
| A21 | A22 | 0.43 | 1 |
| A11 | A23 | 0.74 | 1 |
| A13 | A23 | 0.41 | 1 |
| A15 | A23 | −0.22 | 1 |
| A16 | A23 | 0.25 | 1 |
| A17 | A23 | 0.071 | 1 |
| A21 | A23 | 0.54 | 1 |
| A22 | A23 | 0.8 | 1 |
| A11 | A24 | 0.065 | 1 |
| A13 | A24 | −0.44 | 1 |
| A14 | A24 | −0.44 | 1 |
| A15 | A24 | −0.29 | 1 |
| A16 | A24 | −0.44 | 1 |
| A17 | A24 | −0.57 | 1 |
| A21 | A24 | −0.29 | 1 |
| A22 | A24 | −0.13 | 1 |
| A23 | A24 | −0.43 | 1 |
| A11 | A25 | 0.75 | 1 |
| A13 | A25 | 0.33 | 1 |
| A15 | A25 | −0.22 | 1 |
| A16 | A25 | 0.33 | 1 |
| A17 | A25 | 0.14 | 1 |
| A21 | A25 | 0.65 | 1 |
| A22 | A25 | 0.79 | 1 |
| A24 | A25 | −0.44 | 1 |
| A11 | A26 | 0.75 | 1 |
| A13 | A26 | 0.33 | 1 |
| A15 | A26 | −0.22 | 1 |
| A16 | A26 | 0.33 | 1 |
| A17 | A26 | 0.14 | 1 |
| A21 | A26 | 0.65 | 1 |
| A22 | A26 | 0.79 | 1 |
| A24 | A26 | −0.44 | 1 |
| A11 | B112 | 0.75 | 1 |
| A13 | B112 | 0.33 | 1 |
| A15 | B112 | −0.22 | 1 |
| A16 | B112 | 0.33 | 1 |
| A17 | B112 | 0.14 | 1 |
| A21 | B112 | 0.65 | 1 |
| A22 | B112 | 0.79 | 1 |
| A24 | B112 | −0.44 | 1 |
| A11 | B114 | −0.29 | 1 |
| A13 | B114 | −0.22 | 1 |
| A14 | B114 | −0.22 | 1 |
| A15 | B114 | −0.14 | 1 |
| A16 | B114 | −0.22 | 1 |
| A17 | B114 | −0.28 | 1 |
| A21 | B114 | −0.14 | 1 |
| A22 | B114 | −0.43 | 1 |
| A23 | B114 | −0.22 | 1 |
| A24 | B114 | 0.38 | 1 |
| A25 | B114 | −0.22 | 1 |
| A26 | B114 | −0.22 | 1 |
| B112 | B114 | −0.22 | 1 |
| A11 | B116 | 0.75 | 1 |
| A13 | B116 | −0.33 | 1 |
| A14 | B116 | 0.33 | 1 |
| A15 | B116 | −0.22 | 1 |
| A16 | B116 | 0.33 | 1 |
| A17 | B116 | 0.14 | 1 |
| A21 | B116 | 0.65 | 1 |
| A22 | B116 | 0.4 | 1 |
| A23 | B116 | 0.25 | 1 |
| A24 | B116 | 0.29 | 1 |
| A25 | B116 | 0.33 | 1 |
| A26 | B116 | 0.33 | 1 |
| B112 | B116 | 0.33 | 1 |
| B114 | B116 | −0.22 | 1 |
| A11 | B117 | −0.44 | 1 |
| A13 | B117 | 0.41 | 1 |
| A14 | B117 | −0.33 | 1 |
| A15 | B117 | −0.22 | 1 |
| A16 | B117 | −0.33 | 1 |
| A17 | B117 | 0.054 | 1 |
| A21 | B117 | −0.22 | 1 |
| A22 | B117 | −0.21 | 1 |
| A23 | B117 | −0.33 | 1 |
| A24 | B117 | 0.009 | 1 |
| A25 | B117 | −0.33 | 1 |
| A26 | B117 | −0.33 | 1 |
| B112 | B117 | −0.33 | 1 |
| B114 | B117 | 0.54 | 1 |
| B116 | B117 | −0.33 | 1 |
| A11 | B121 | 0.75 | 1 |
| A13 | B121 | 0.33 | 1 |
| A15 | B121 | −0.22 | 1 |
| A16 | B121 | 0.33 | 1 |
| A17 | B121 | 0.14 | 1 |
| A21 | B121 | 0.65 | 1 |
| A22 | B121 | 0.79 | 1 |
| A24 | B121 | −0.44 | 1 |
| B114 | B121 | −0.22 | 1 |
| B116 | B121 | 0.33 | 1 |
| B117 | B121 | −0.33 | 1 |
| A11 | B122 | 0.15 | 1 |
| A13 | B122 | −0.33 | 1 |
| A14 | B122 | 0.33 | 1 |
| A15 | B122 | −0.22 | 1 |
| A16 | B122 | 0.33 | 1 |
| A17 | B122 | 0.14 | 1 |
| A21 | B122 | 0.65 | 1 |
| A22 | B122 | 0.4 | 1 |
| A23 | B122 | 0.25 | 1 |
| A24 | B122 | 0.073 | 1 |
| A25 | B122 | 0.33 | 1 |
| A26 | B122 | 0.33 | 1 |
| B112 | B122 | 0.33 | 1 |
| B114 | B122 | −0.22 | 1 |
| B116 | B122 | 0.33 | 1 |
| B117 | B122 | −0.33 | 1 |
| B121 | B122 | 0.33 | 1 |
| A11 | B123 | −0.29 | 1 |
| A13 | B123 | 0.65 | 1 |
| A14 | B123 | −0.22 | 1 |
| A15 | B123 | −0.14 | 1 |
| A16 | B123 | −0.22 | 1 |
| A17 | B123 | 0.28 | 1 |
| A21 | B123 | −0.14 | 1 |
| A22 | B123 | 0.087 | 1 |
| A23 | B123 | −0.22 | 1 |
| A24 | B123 | −0.29 | 1 |
| A25 | B123 | −0.22 | 1 |
| A26 | B123 | −0.22 | 1 |
| B112 | B123 | −0.22 | 1 |
| B114 | B123 | −0.14 | 1 |
| B116 | B123 | −0.22 | 1 |
| B117 | B123 | 0.76 | 1 |
| B121 | B123 | −0.22 | 1 |
| B122 | B123 | −0.22 | 1 |
| A11 | B124 | 0.49 | 1 |
| A13 | B124 | 0.65 | 1 |
| A14 | B124 | 0.65 | 1 |
| A15 | B124 | −0.14 | 1 |
| A16 | B124 | −0.22 | 1 |
| A17 | B124 | −0.28 | 1 |
| A21 | B124 | −0.14 | 1 |
| A22 | B124 | 0.61 | 1 |
| A23 | B124 | 0.76 | 1 |
| A24 | B124 | −0.29 | 1 |
| A25 | B124 | 0.65 | 1 |
| A26 | B124 | 0.65 | 1 |
| B112 | B124 | 0.65 | 1 |
| B114 | B124 | −0.14 | 1 |
| B116 | B124 | −0.22 | 1 |
| B117 | B124 | −0.22 | 1 |
| B121 | B124 | 0.65 | 1 |
| B122 | B124 | −0.22 | 1 |
| B123 | B124 | −0.14 | 1 |
| A11 | B125 | 0.15 | 1 |
| A14 | B125 | 0.33 | 1 |
| A15 | B125 | −0.22 | 1 |
| A16 | B125 | −0.33 | 1 |
| A17 | B125 | 0 | 1 |
| A21 | B125 | −0.22 | 1 |
| A22 | B125 | 0.53 | 1 |
| A23 | B125 | 0.41 | 1 |
| A24 | B125 | −0.44 | 1 |
| A25 | B125 | 0.33 | 1 |
| A26 | B125 | 0.33 | 1 |
| B112 | B125 | 0.33 | 1 |
| B114 | B125 | −0.22 | 1 |
| B116 | B125 | −0.33 | 1 |
| B117 | B125 | 0.41 | 1 |
| B121 | B125 | 0.33 | 1 |
| B122 | B125 | −0.33 | 1 |
| B123 | B125 | 0.65 | 1 |
| B124 | B125 | 0.65 | 1 |
| A11 | B126 | 0 | 1 |
| A13 | B126 | 0.14 | 1 |
| A14 | B126 | −0.14 | 1 |
| A15 | B126 | −0.36 | 1 |
| A16 | B126 | −0.14 | 1 |
| A17 | B126 | 0.088 | 1 |
| A21 | B126 | 0.18 | 1 |
| A22 | B126 | −0.027 | 1 |
| A23 | B126 | −0.19 | 1 |
| A24 | B126 | 0.25 | 1 |
| A25 | B126 | −0.14 | 1 |
| A26 | B126 | −0.14 | 1 |
| B112 | B126 | −0.14 | 1 |
| B114 | B126 | 0.53 | 1 |
| B116 | B126 | 0.27 | 1 |
| B117 | B126 | 0.81 | 1 |
| B121 | B126 | −0.14 | 1 |
| B122 | B126 | −0.14 | 1 |
| B123 | B126 | 0.53 | 1 |
| B124 | B126 | −0.36 | 1 |
| B125 | B126 | 0.14 | 1 |
| A11 | B127 | 0.49 | 1 |
| A13 | B127 | −0.22 | 1 |
| A14 | B127 | 0.65 | 1 |
| A15 | B127 | −0.14 | 1 |
| A16 | B127 | 0.65 | 1 |
| A17 | B127 | 0.47 | 1 |
| A22 | B127 | 0.43 | 1 |
| A23 | B127 | 0.54 | 1 |
| A24 | B127 | −0.29 | 1 |
| A25 | B127 | 0.65 | 1 |
| A26 | B127 | 0.65 | 1 |
| B112 | B127 | 0.65 | 1 |
| B114 | B127 | −0.14 | 1 |
| B116 | B127 | 0.65 | 1 |
| B117 | B127 | −0.22 | 1 |
| B121 | B127 | 0.65 | 1 |
| B122 | B127 | 0.65 | 1 |
| B123 | B127 | −0.14 | 1 |
| B124 | B127 | −0.14 | 1 |
| B125 | B127 | −0.22 | 1 |
| B126 | B127 | 0.18 | 1 |
| A11 | B211 | 0.56 | 1 |
| A13 | B211 | 0.69 | 1 |
| A14 | B211 | 0.41 | 1 |
| A15 | B211 | −0.36 | 1 |
| A16 | B211 | −0.069 | 1 |
| A17 | B211 | 0.19 | 1 |
| A21 | B211 | 0.27 | 1 |
| A22 | B211 | 0.7 | 1 |
| A23 | B211 | 0.41 | 1 |
| A24 | B211 | −0.20 | 1 |
| A25 | B211 | 0.41 | 1 |
| A26 | B211 | 0.41 | 1 |
| B112 | B211 | 0.41 | 1 |
| B114 | B211 | −0.36 | 1 |
| B116 | B211 | 0.41 | 1 |
| B117 | B211 | 0.3 | 1 |
| B121 | B211 | 0.41 | 1 |
| B122 | B211 | −0.069 | 1 |
| B123 | B211 | 0.63 | 1 |
| B124 | B211 | 0.27 | 1 |
| B125 | B211 | 0.69 | 1 |
| B126 | B211 | 0.46 | 1 |
| B127 | B211 | 0.27 | 1 |
| A11 | B212 | −0.45 | 1 |
| A13 | B212 | 0.33 | 1 |
| A14 | B212 | −0.33 | 1 |
| A15 | B212 | 0.65 | 1 |
| A16 | B212 | 0.33 | 1 |
| A17 | B212 | 0.72 | 1 |
| A21 | B212 | −0.22 | 1 |
| A22 | B212 | −0.26 | 1 |
| A23 | B212 | −0.33 | 1 |
| A24 | B212 | −0.44 | 1 |
| A25 | B212 | −0.33 | 1 |
| A26 | B212 | −0.33 | 1 |
| B112 | B212 | −0.33 | 1 |
| B114 | B212 | −0.22 | 1 |
| B116 | B212 | −0.33 | 1 |
| B117 | B212 | 0.41 | 1 |
| B121 | B212 | −0.33 | 1 |
| B122 | B212 | −0.33 | 1 |
| B123 | B212 | 0.65 | 1 |
| B124 | B212 | −0.22 | 1 |
| B125 | B212 | 0.33 | 1 |
| B126 | B212 | 0.14 | 1 |
| B127 | B212 | −0.22 | 1 |
| B211 | B212 | 0.21 | 1 |
| A11 | B213 | −0.29 | 1 |
| A13 | B213 | −0.22 | 1 |
| A14 | B213 | −0.22 | 1 |
| A15 | B213 | −0.14 | 1 |
| A16 | B213 | −0.22 | 1 |
| A17 | B213 | −0.28 | 1 |
| A21 | B213 | −0.14 | 1 |
| A22 | B213 | 0.087 | 1 |
| A23 | B213 | −0.22 | 1 |
| A24 | B213 | 0.38 | 1 |
| A25 | B213 | −0.22 | 1 |
| A26 | B213 | −0.22 | 1 |
| B112 | B213 | −0.22 | 1 |
| B114 | B213 | −0.14 | 1 |
| B116 | B213 | −0.22 | 1 |
| B117 | B213 | −0.22 | 1 |
| B121 | B213 | −0.22 | 1 |
| B122 | B213 | 0.65 | 1 |
| B123 | B213 | −0.14 | 1 |
| B124 | B213 | −0.14 | 1 |
| B125 | B213 | −0.22 | 1 |
| B126 | B213 | −0.36 | 1 |
| B127 | B213 | −0.14 | 1 |
| B211 | B213 | −0.36 | 1 |
| B212 | B213 | −0.22 | 1 |
| A11 | B214 | −0.29 | 1 |
| A13 | B214 | −0.22 | 1 |
| A14 | B214 | −0.22 | 1 |
| A16 | B214 | 0.65 | 1 |
| A17 | B214 | 0.66 | 1 |
| A21 | B214 | −0.14 | 1 |
| A22 | B214 | −0.43 | 1 |
| A23 | B214 | −0.22 | 1 |
| A24 | B214 | −0.29 | 1 |
| A25 | B214 | −0.22 | 1 |
| A26 | B214 | −0.22 | 1 |
| B112 | B214 | −0.22 | 1 |
| B114 | B214 | −0.14 | 1 |
| B116 | B214 | −0.22 | 1 |
| B117 | B214 | −0.22 | 1 |
| B121 | B214 | −0.22 | 1 |
| B122 | B214 | −0.22 | 1 |
| B123 | B214 | −0.14 | 1 |
| B124 | B214 | −0.14 | 1 |
| B125 | B214 | −0.22 | 1 |
| B126 | B214 | −0.36 | 1 |
| B127 | B214 | −0.14 | 1 |
| B211 | B214 | −0.36 | 1 |
| B212 | B214 | 0.65 | 1 |
| B213 | B214 | −0.14 | 1 |
| A11 | B215 | 0.24 | 1 |
| A13 | B215 | 0.27 | 1 |
| A14 | B215 | 0.54 | 1 |
| A15 | B215 | 0.53 | 1 |
| A16 | B215 | 0.82 | 1 |
| A17 | B215 | 0.88 | 1 |
| A21 | B215 | 0.53 | 1 |
| A22 | B215 | 0.32 | 1 |
| A23 | B215 | 0.51 | 1 |
| A24 | B215 | −0.71 | 1 |
| A25 | B215 | 0.54 | 1 |
| A26 | B215 | 0.54 | 1 |
| B112 | B215 | 0.54 | 1 |
| B114 | B215 | −0.36 | 1 |
| B116 | B215 | 0.14 | 1 |
| B117 | B215 | −0.084 | 1 |
| B121 | B215 | 0.54 | 1 |
| B122 | B215 | 0.14 | 1 |
| B123 | B215 | 0.18 | 1 |
| B124 | B215 | 0.18 | 1 |
| B125 | B215 | 0.27 | 1 |
| B126 | B215 | −0.056 | 1 |
| B127 | B215 | 0.53 | 1 |
| B211 | B215 | 0.35 | 1 |
| B212 | B215 | 0.54 | 1 |
| B213 | B215 | −0.36 | 1 |
| B214 | B215 | 0.53 | 1 |
| A11 | B217 | −0.29 | 1 |
| A13 | B217 | 0.65 | 1 |
| A14 | B217 | −0.22 | 1 |
| A15 | B217 | −0.14 | 1 |
| A16 | B217 | −0.22 | 1 |
| A17 | B217 | 0.28 | 1 |
| A21 | B217 | −0.14 | 1 |
| A22 | B217 | 0.087 | 1 |
| A23 | B217 | −0.22 | 1 |
| A24 | B217 | −0.29 | 1 |
| A25 | B217 | −0.22 | 1 |
| A26 | B217 | −0.22 | 1 |
| B112 | B217 | −0.22 | 1 |
| B114 | B217 | −0.14 | 1 |
| B116 | B217 | −0.22 | 1 |
| B117 | B217 | 0.76 | 1 |
| B121 | B217 | −0.22 | 1 |
| B122 | B217 | −0.22 | 1 |
| B124 | B217 | −0.14 | 1 |
| B125 | B217 | 0.65 | 1 |
| B126 | B217 | 0.53 | 1 |
| B127 | B217 | −0.14 | 1 |
| B211 | B217 | 0.63 | 1 |
| B212 | B217 | 0.65 | 1 |
| B213 | B217 | −0.14 | 1 |
| B214 | B217 | −0.14 | 1 |
| B215 | B217 | 0.18 | 1 |
| A11 | B221 | −0.13 | 1 |
| A13 | B221 | 0.29 | 1 |
| A14 | B221 | 0.073 | 1 |
| A15 | B221 | 0.38 | 1 |
| A16 | B221 | 0.58 | 1 |
| A17 | B221 | 0.9 | 1 |
| A21 | B221 | 0.38 | 1 |
| A22 | B221 | 0.072 | 1 |
| A23 | B221 | 0.009 | 1 |
| A24 | B221 | −0.57 | 1 |
| A25 | B221 | 0.073 | 1 |
| A26 | B221 | 0.073 | 1 |
| B112 | B221 | 0.073 | 1 |
| B114 | B221 | −0.29 | 1 |
| B116 | B221 | 0.073 | 1 |
| B117 | B221 | 0.38 | 1 |
| B121 | B221 | 0.073 | 1 |
| B122 | B221 | 0.073 | 1 |
| B123 | B221 | 0.67 | 1 |
| B124 | B221 | −0.29 | 1 |
| B125 | B221 | 0.29 | 1 |
| B126 | B221 | 0.34 | 1 |
| B127 | B221 | 0.38 | 1 |
| B211 | B221 | 0.47 | 1 |
| B212 | B221 | 0.8 | 1 |
| B213 | B221 | −0.29 | 1 |
| B214 | B221 | 0.38 | 1 |
| B215 | B221 | 0.77 | 1 |
| B217 | B221 | 0.67 | 1 |
| A11 | B222 | −0.29 | 1 |
| A13 | B222 | −0.22 | 1 |
| A14 | B222 | −0.22 | 1 |
| A15 | B222 | −0.14 | 1 |
| A16 | B222 | −0.22 | 1 |
| A17 | B222 | −0.28 | 1 |
| A21 | B222 | −0.14 | 1 |
| A22 | B222 | 0.087 | 1 |
| A23 | B222 | −0.22 | 1 |
| A24 | B222 | 0.38 | 1 |
| A25 | B222 | −0.22 | 1 |
| A26 | B222 | −0.22 | 1 |
| B112 | B222 | −0.22 | 1 |
| B114 | B222 | −0.14 | 1 |
| B116 | B222 | −0.22 | 1 |
| B117 | B222 | −0.22 | 1 |
| B121 | B222 | −0.22 | 1 |
| B122 | B222 | 0.65 | 1 |
| B123 | B222 | −0.14 | 1 |
| B124 | B222 | −0.14 | 1 |
| B125 | B222 | −0.22 | 1 |
| B126 | B222 | −0.36 | 1 |
| B127 | B222 | −0.14 | 1 |
| B211 | B222 | −0.36 | 1 |
| B212 | B222 | −0.22 | 1 |
| B214 | B222 | −0.14 | 1 |
| B215 | B222 | −0.36 | 1 |
| B217 | B222 | −0.14 | 1 |
| B221 | B222 | −0.29 | 1 |
| A11 | B223 | −0.44 | 1 |
| A13 | B223 | 0.41 | 1 |
| A14 | B223 | −0.33 | 1 |
| A15 | B223 | −0.22 | 1 |
| A16 | B223 | −0.33 | 1 |
| A17 | B223 | 0.054 | 1 |
| A21 | B223 | −0.22 | 1 |
| A22 | B223 | 0.13 | 1 |
| A23 | B223 | −0.33 | 1 |
| A24 | B223 | 0.009 | 1 |
| A25 | B223 | −0.33 | 1 |
| A26 | B223 | −0.33 | 1 |
| B112 | B223 | −0.33 | 1 |
| B114 | B223 | −0.22 | 1 |
| B116 | B223 | −0.33 | 1 |
| B117 | B223 | 0.5 | 1 |
| B121 | B223 | −0.33 | 1 |
| B122 | B223 | 0.25 | 1 |
| B123 | B223 | 0.76 | 1 |
| B124 | B223 | −0.22 | 1 |
| B125 | B223 | 0.41 | 1 |
| B126 | B223 | 0.22 | 1 |
| B127 | B223 | −0.22 | 1 |
| B211 | B223 | 0.3 | 1 |
| B212 | B223 | 0.41 | 1 |
| B213 | B223 | 0.54 | 1 |
| B214 | B223 | −0.22 | 1 |
| B215 | B223 | −0.084 | 1 |
| B217 | B223 | 0.76 | 1 |
| B221 | B223 | 0.38 | 1 |
| B222 | B223 | 0.54 | 1 |
| A11 | B224 | 0.56 | 1 |
| A13 | B224 | −0.069 | 1 |
| A14 | B224 | 0.41 | 1 |
| A15 | B224 | 0.63 | 1 |
| A16 | B224 | 0.69 | 1 |
| A17 | B224 | 0.52 | 1 |
| A21 | B224 | 0.27 | 1 |
| A22 | B224 | 0.25 | 1 |
| A23 | B224 | 0.41 | 1 |
| A24 | B224 | −0.20 | 1 |
| A25 | B224 | 0.41 | 1 |
| A26 | B224 | 0.41 | 1 |
| B112 | B224 | 0.41 | 1 |
| B114 | B224 | −0.36 | 1 |
| B116 | B224 | 0.41 | 1 |
| B117 | B224 | −0.55 | 1 |
| B121 | B224 | 0.41 | 1 |
| B122 | B224 | −0.069 | 1 |
| B123 | B224 | −0.36 | 1 |
| B124 | B224 | 0.27 | 1 |
| B125 | B224 | −0.069 | 1 |
| B126 | B224 | −0.31 | 1 |
| B127 | B224 | 0.27 | 1 |
| B211 | B224 | 0.14 | 1 |
| B212 | B224 | 0.21 | 1 |
| B213 | B224 | −0.36 | 1 |
| B214 | B224 | 0.63 | 1 |
| B215 | B224 | 0.66 | 1 |
| B217 | B224 | −0.36 | 1 |
| B221 | B224 | 0.23 | 1 |
| B222 | B224 | −0.36 | 1 |
| B223 | B224 | −0.55 | 1 |
| A11 | B225 | −0.44 | 1 |
| A13 | B225 | 0.25 | 1 |
| A14 | B225 | −0.33 | 1 |
| A15 | B225 | −0.22 | 1 |
| A16 | B225 | −0.33 | 1 |
| A17 | B225 | −0.054 | 1 |
| A21 | B225 | −0.22 | 1 |
| A22 | B225 | −0.31 | 1 |
| A23 | B225 | −0.33 | 1 |
| A24 | B225 | 0.13 | 1 |
| A25 | B225 | −0.33 | 1 |
| A26 | B225 | −0.33 | 1 |
| B112 | B225 | −0.33 | 1 |
| B114 | B225 | 0.76 | 1 |
| B116 | B225 | −0.33 | 1 |
| B121 | B225 | −0.33 | 1 |
| B122 | B225 | −0.33 | 1 |
| B123 | B225 | 0.54 | 1 |
| B124 | B225 | −0.22 | 1 |
| B125 | B225 | 0.25 | 1 |
| B126 | B225 | 0.81 | 1 |
| B127 | B225 | −0.22 | 1 |
| B211 | B225 | 0.11 | 1 |
| B212 | B225 | 0.25 | 1 |
| B213 | B225 | −0.22 | 1 |
| B214 | B225 | −0.22 | 1 |
| B215 | B225 | −0.19 | 1 |
| B217 | B225 | 0.54 | 1 |
| B221 | B225 | 0.2 | 1 |
| B222 | B225 | −0.22 | 1 |
| B223 | B225 | 0.32 | 1 |
| B224 | B225 | −0.55 | 1 |
| A11 | B226 | 0.15 | 1 |
| A13 | B226 | 0.33 | 1 |
| A14 | B226 | 0.33 | 1 |
| A15 | B226 | −0.22 | 1 |
| A16 | B226 | 0.33 | 1 |
| A17 | B226 | 0.58 | 1 |
| A21 | B226 | 0.65 | 1 |
| A22 | B226 | 0.4 | 1 |
| A23 | B226 | 0.25 | 1 |
| A24 | B226 | −0.44 | 1 |
| A25 | B226 | 0.33 | 1 |
| A26 | B226 | 0.33 | 1 |
| B112 | B226 | 0.33 | 1 |
| B114 | B226 | −0.22 | 1 |
| B116 | B226 | 0.33 | 1 |
| B117 | B226 | 0.41 | 1 |
| B121 | B226 | 0.33 | 1 |
| B122 | B226 | 0.33 | 1 |
| B123 | B226 | 0.65 | 1 |
| B124 | B226 | −0.22 | 1 |
| B125 | B226 | 0.33 | 1 |
| B126 | B226 | 0.54 | 1 |
| B127 | B226 | 0.65 | 1 |
| B211 | B226 | 0.69 | 1 |
| B212 | B226 | 0.33 | 1 |
| B213 | B226 | −0.22 | 1 |
| B214 | B226 | −0.22 | 1 |
| B215 | B226 | 0.54 | 1 |
| B217 | B226 | 0.65 | 1 |
| B221 | B226 | 0.8 | 1 |
| B222 | B226 | −0.22 | 1 |
| B223 | B226 | 0.41 | 1 |
| B224 | B226 | −0.069 | 1 |
| B225 | B226 | 0.25 | 1 |
| A11 | B227 | 0.4 | 1 |
| A13 | B227 | 0 | 1 |
| A14 | B227 | 0.58 | 1 |
| A15 | B227 | 0.42 | 1 |
| A16 | B227 | 0.77 | 1 |
| A17 | B227 | 0.65 | 1 |
| A21 | B227 | 0.59 | 1 |
| A22 | B227 | 0.22 | 1 |
| A23 | B227 | 0.53 | 1 |
| A24 | B227 | −0.34 | 1 |
| A25 | B227 | 0.58 | 1 |
| A26 | B227 | 0.58 | 1 |
| B112 | B227 | 0.58 | 1 |
| B114 | B227 | 0.17 | 1 |
| B116 | B227 | 0.32 | 1 |
| B117 | B227 | −0.032 | 1 |
| B121 | B227 | 0.58 | 1 |
| B122 | B227 | 0.064 | 1 |
| B123 | B227 | −0.17 | 1 |
| B124 | B227 | 0.17 | 1 |
| B125 | B227 | 0 | 1 |
| B126 | B227 | 0.2 | 1 |
| B127 | B227 | 0.59 | 1 |
| B211 | B227 | 0.18 | 1 |
| B212 | B227 | 0.19 | 1 |
| B213 | B227 | −0.50 | 1 |
| B214 | B227 | 0.42 | 1 |
| B215 | B227 | 0.79 | 1 |
| B217 | B227 | −0.17 | 1 |
| B221 | B227 | 0.45 | 1 |
| B222 | B227 | −0.50 | 1 |
| B223 | B227 | −0.48 | 1 |
| B224 | B227 | 0.69 | 1 |
| B225 | B227 | 0.032 | 1 |
| B226 | B227 | 0.32 | 1 |

| **Supplementary Table S4.** Pairwise assessment of independence of trap loctions in 2024 during a field trapping experiment for *E. giganteana* in *Silphium integrifolium* fields in Salina, KS using Spearman ρ correlations. Warmer colors (=red) indicate lower P-values, while cooler colors (=blue) indicate higher P-values. | | | |
| --- | --- | --- | --- |
| **Pairwise Comparison** | | *ρ* | *P* |
| Trap Location 1 | Trap Location 2 |  |  |
| B321 | B331 | 1 | 0.0001 |
| B312 | C11 | 1 | 0.0001 |
| B333 | C14 | 1 | 0.0001 |
| B333 | C21 | 1 | 0.0001 |
| C14 | C21 | 1 | 0.0001 |
| C23 | C32 | 1 | 0.0001 |
| B313 | C33 | 1 | 0.0001 |
| B312 | D12 | 1 | 0.0001 |
| C11 | D12 | 1 | 0.0001 |
| B312 | D23 | 1 | 0.0001 |
| C11 | D23 | 1 | 0.0001 |
| D12 | D23 | 1 | 0.0001 |
| B333 | D24 | 1 | 0.0001 |
| C14 | D24 | 1 | 0.0001 |
| C21 | D24 | 1 | 0.0001 |
| D22 | D31 | 1 | 0.0001 |
| D21 | D32 | 1 | 0.0001 |
| C23 | D13 | 0.99 | 0.0013 |
| C32 | D13 | 0.99 | 0.0013 |
| B311 | C31 | 0.98 | 0.016 |
| B322 | C12 | 0.94 | 0.2 |
| B332 | C22 | 0.93 | 0.48 |
| B311 | B312 | −0.25 | 1 |
| B311 | B313 | 0.33 | 1 |
| B312 | B313 | 0.65 | 1 |
| B311 | B314 | 0.44 | 1 |
| B312 | B314 | −0.25 | 1 |
| B313 | B314 | 0.38 | 1 |
| B311 | B321 | 0.57 | 1 |
| B312 | B321 | −0.12 | 1 |
| B313 | B321 | −0.25 | 1 |
| B314 | B321 | −0.25 | 1 |
| B311 | B322 | −0.15 | 1 |
| B312 | B322 | 0.41 | 1 |
| B313 | B322 | 0.42 | 1 |
| B314 | B322 | 0.55 | 1 |
| B321 | B322 | −0.25 | 1 |
| B311 | B323 | −0.15 | 1 |
| B312 | B323 | 0.65 | 1 |
| B313 | B323 | 0.62 | 1 |
| B314 | B323 | −0.055 | 1 |
| B321 | B323 | −0.25 | 1 |
| B322 | B323 | 0.42 | 1 |
| B311 | B331 | 0.57 | 1 |
| B312 | B331 | −0.12 | 1 |
| B313 | B331 | −0.25 | 1 |
| B314 | B331 | −0.25 | 1 |
| B322 | B331 | −0.25 | 1 |
| B323 | B331 | −0.25 | 1 |
| B311 | B332 | 0.055 | 1 |
| B312 | B332 | 0.5 | 1 |
| B313 | B332 | 0.55 | 1 |
| B314 | B332 | 0.5 | 1 |
| B321 | B332 | −0.25 | 1 |
| B322 | B332 | 0.55 | 1 |
| B323 | B332 | 0.11 | 1 |
| B331 | B332 | −0.25 | 1 |
| B311 | B333 | 0.33 | 1 |
| B312 | B333 | −0.12 | 1 |
| B313 | B333 | 0.41 | 1 |
| B314 | B333 | 0.5 | 1 |
| B321 | B333 | −0.12 | 1 |
| B322 | B333 | 0.41 | 1 |
| B323 | B333 | 0.41 | 1 |
| B331 | B333 | −0.12 | 1 |
| B332 | B333 | −0.25 | 1 |
| B311 | C11 | −0.29 | 1 |
| B313 | C11 | 0.67 | 1 |
| B314 | C11 | −0.29 | 1 |
| B321 | C11 | −0.14 | 1 |
| B322 | C11 | 0.38 | 1 |
| B323 | C11 | 0.67 | 1 |
| B331 | C11 | −0.14 | 1 |
| B332 | C11 | 0.49 | 1 |
| B333 | C11 | −0.14 | 1 |
| B311 | C12 | −0.28 | 1 |
| B312 | C12 | 0.66 | 1 |
| B313 | C12 | 0.55 | 1 |
| B314 | C12 | 0.32 | 1 |
| B321 | C12 | −0.28 | 1 |
| B323 | C12 | 0.55 | 1 |
| B331 | C12 | −0.28 | 1 |
| B332 | C12 | 0.58 | 1 |
| B333 | C12 | 0.28 | 1 |
| C11 | C12 | 0.66 | 1 |
| B311 | C13 | −0.20 | 1 |
| B312 | C13 | 0.49 | 1 |
| B313 | C13 | 0.52 | 1 |
| B314 | C13 | −0.067 | 1 |
| B321 | C13 | −0.29 | 1 |
| B322 | C13 | 0.33 | 1 |
| B323 | C13 | 0.52 | 1 |
| B331 | C13 | −0.29 | 1 |
| B332 | C13 | −0.067 | 1 |
| B333 | C13 | 0.49 | 1 |
| C11 | C13 | 0.49 | 1 |
| C12 | C13 | 0.45 | 1 |
| B311 | C14 | 0.29 | 1 |
| B312 | C14 | −0.14 | 1 |
| B313 | C14 | 0.38 | 1 |
| B314 | C14 | 0.49 | 1 |
| B321 | C14 | −0.14 | 1 |
| B322 | C14 | 0.38 | 1 |
| B323 | C14 | 0.38 | 1 |
| B331 | C14 | −0.14 | 1 |
| B332 | C14 | −0.29 | 1 |
| C11 | C14 | −0.14 | 1 |
| C12 | C14 | 0.28 | 1 |
| C13 | C14 | 0.49 | 1 |
| B311 | C21 | 0.29 | 1 |
| B312 | C21 | −0.14 | 1 |
| B313 | C21 | 0.38 | 1 |
| B314 | C21 | 0.49 | 1 |
| B321 | C21 | −0.14 | 1 |
| B322 | C21 | 0.38 | 1 |
| B323 | C21 | 0.38 | 1 |
| B331 | C21 | −0.14 | 1 |
| B332 | C21 | −0.29 | 1 |
| C11 | C21 | −0.14 | 1 |
| C12 | C21 | 0.28 | 1 |
| C13 | C21 | 0.49 | 1 |
| B311 | C22 | 0.11 | 1 |
| B312 | C22 | 0.45 | 1 |
| B313 | C22 | 0.69 | 1 |
| B314 | C22 | 0.68 | 1 |
| B321 | C22 | −0.36 | 1 |
| B322 | C22 | 0.69 | 1 |
| B323 | C22 | 0.22 | 1 |
| B331 | C22 | −0.36 | 1 |
| B333 | C22 | 0.09 | 1 |
| C11 | C22 | 0.45 | 1 |
| C12 | C22 | 0.72 | 1 |
| C13 | C22 | 0.12 | 1 |
| C14 | C22 | 0.09 | 1 |
| C21 | C22 | 0.09 | 1 |
| B311 | C23 | 0 | 1 |
| B312 | C23 | 0.65 | 1 |
| B313 | C23 | 0.8 | 1 |
| B314 | C23 | 0.15 | 1 |
| B321 | C23 | −0.22 | 1 |
| B322 | C23 | 0.58 | 1 |
| B323 | C23 | 0.8 | 1 |
| B331 | C23 | −0.22 | 1 |
| B332 | C23 | 0.15 | 1 |
| B333 | C23 | 0.65 | 1 |
| C11 | C23 | 0.65 | 1 |
| C12 | C23 | 0.72 | 1 |
| C13 | C23 | 0.75 | 1 |
| C14 | C23 | 0.65 | 1 |
| C21 | C23 | 0.65 | 1 |
| C22 | C23 | 0.41 | 1 |
| B311 | C24 | 0 | 1 |
| B312 | C24 | −0.22 | 1 |
| B313 | C24 | 0.073 | 1 |
| B314 | C24 | 0.75 | 1 |
| B321 | C24 | −0.22 | 1 |
| B322 | C24 | 0.8 | 1 |
| B323 | C24 | 0.073 | 1 |
| B331 | C24 | −0.22 | 1 |
| B332 | C24 | 0.15 | 1 |
| B333 | C24 | 0.65 | 1 |
| C11 | C24 | −0.22 | 1 |
| C12 | C24 | 0.58 | 1 |
| C13 | C24 | 0.15 | 1 |
| C14 | C24 | 0.65 | 1 |
| C21 | C24 | 0.65 | 1 |
| C22 | C24 | 0.41 | 1 |
| C23 | C24 | 0.33 | 1 |
| B312 | C31 | −0.29 | 1 |
| B313 | C31 | 0.21 | 1 |
| B314 | C31 | 0.33 | 1 |
| B321 | C31 | 0.67 | 1 |
| B322 | C31 | −0.18 | 1 |
| B323 | C31 | −0.18 | 1 |
| B331 | C31 | 0.67 | 1 |
| B332 | C31 | −0.13 | 1 |
| B333 | C31 | 0.38 | 1 |
| C11 | C31 | −0.29 | 1 |
| C12 | C31 | −0.24 | 1 |
| C13 | C31 | −0.13 | 1 |
| C14 | C31 | 0.38 | 1 |
| C21 | C31 | 0.38 | 1 |
| C22 | C31 | 0.015 | 1 |
| C23 | C31 | 0.073 | 1 |
| C24 | C31 | 0.073 | 1 |
| B311 | C32 | 0 | 1 |
| B312 | C32 | 0.65 | 1 |
| B313 | C32 | 0.8 | 1 |
| B314 | C32 | 0.15 | 1 |
| B321 | C32 | −0.22 | 1 |
| B322 | C32 | 0.58 | 1 |
| B323 | C32 | 0.8 | 1 |
| B331 | C32 | −0.22 | 1 |
| B332 | C32 | 0.15 | 1 |
| B333 | C32 | 0.65 | 1 |
| C11 | C32 | 0.65 | 1 |
| C12 | C32 | 0.72 | 1 |
| C13 | C32 | 0.75 | 1 |
| C14 | C32 | 0.65 | 1 |
| C21 | C32 | 0.65 | 1 |
| C22 | C32 | 0.41 | 1 |
| C24 | C32 | 0.33 | 1 |
| C31 | C32 | 0.073 | 1 |
| B311 | C33 | 0.26 | 1 |
| B312 | C33 | 0.67 | 1 |
| B314 | C33 | 0.33 | 1 |
| B321 | C33 | −0.29 | 1 |
| B322 | C33 | 0.37 | 1 |
| B323 | C33 | 0.61 | 1 |
| B331 | C33 | −0.29 | 1 |
| B332 | C33 | 0.52 | 1 |
| B333 | C33 | 0.38 | 1 |
| C11 | C33 | 0.67 | 1 |
| C12 | C33 | 0.55 | 1 |
| C13 | C33 | 0.52 | 1 |
| C14 | C33 | 0.38 | 1 |
| C21 | C33 | 0.38 | 1 |
| C22 | C33 | 0.69 | 1 |
| C23 | C33 | 0.8 | 1 |
| C24 | C33 | 0.073 | 1 |
| C31 | C33 | 0.21 | 1 |
| C32 | C33 | 0.8 | 1 |
| B311 | C34 | −0.24 | 1 |
| B312 | C34 | 0.38 | 1 |
| B313 | C34 | 0.37 | 1 |
| B314 | C34 | −0.13 | 1 |
| B321 | C34 | −0.29 | 1 |
| B322 | C34 | 0.21 | 1 |
| B323 | C34 | 0.37 | 1 |
| B331 | C34 | −0.29 | 1 |
| B332 | C34 | −0.13 | 1 |
| B333 | C34 | 0.38 | 1 |
| C11 | C34 | 0.38 | 1 |
| C12 | C34 | 0.31 | 1 |
| C13 | C34 | 0.33 | 1 |
| C14 | C34 | 0.38 | 1 |
| C21 | C34 | 0.38 | 1 |
| C22 | C34 | 0.015 | 1 |
| C23 | C34 | 0.58 | 1 |
| C24 | C34 | 0.073 | 1 |
| C31 | C34 | −0.18 | 1 |
| C32 | C34 | 0.58 | 1 |
| C33 | C34 | 0.37 | 1 |
| B311 | D12 | −0.25 | 1 |
| B313 | D12 | 0.65 | 1 |
| B314 | D12 | −0.25 | 1 |
| B321 | D12 | −0.12 | 1 |
| B322 | D12 | 0.41 | 1 |
| B323 | D12 | 0.65 | 1 |
| B331 | D12 | −0.12 | 1 |
| B332 | D12 | 0.5 | 1 |
| B333 | D12 | −0.12 | 1 |
| C12 | D12 | 0.66 | 1 |
| C13 | D12 | 0.49 | 1 |
| C14 | D12 | −0.14 | 1 |
| C21 | D12 | −0.14 | 1 |
| C22 | D12 | 0.45 | 1 |
| C23 | D12 | 0.65 | 1 |
| C24 | D12 | −0.22 | 1 |
| C31 | D12 | −0.29 | 1 |
| C32 | D12 | 0.65 | 1 |
| C33 | D12 | 0.67 | 1 |
| C34 | D12 | 0.38 | 1 |
| B311 | D13 | 0.014 | 1 |
| B312 | D13 | 0.75 | 1 |
| B313 | D13 | 0.82 | 1 |
| B314 | D13 | 0.12 | 1 |
| B321 | D13 | −0.19 | 1 |
| B322 | D13 | 0.61 | 1 |
| B323 | D13 | 0.82 | 1 |
| B331 | D13 | −0.19 | 1 |
| B332 | D13 | 0.25 | 1 |
| B333 | D13 | 0.56 | 1 |
| C11 | D13 | 0.76 | 1 |
| C12 | D13 | 0.75 | 1 |
| C13 | D13 | 0.74 | 1 |
| C14 | D13 | 0.54 | 1 |
| C21 | D13 | 0.54 | 1 |
| C22 | D13 | 0.44 | 1 |
| C24 | D13 | 0.25 | 1 |
| C31 | D13 | 0.009 | 1 |
| C33 | D13 | 0.82 | 1 |
| C34 | D13 | 0.58 | 1 |
| D12 | D13 | 0.75 | 1 |
| B311 | D14 | −0.25 | 1 |
| B312 | D14 | −0.12 | 1 |
| B313 | D14 | −0.25 | 1 |
| B314 | D14 | −0.25 | 1 |
| B321 | D14 | −0.12 | 1 |
| B322 | D14 | −0.25 | 1 |
| B323 | D14 | −0.25 | 1 |
| B331 | D14 | −0.12 | 1 |
| B332 | D14 | −0.25 | 1 |
| B333 | D14 | −0.12 | 1 |
| C11 | D14 | −0.14 | 1 |
| C12 | D14 | −0.28 | 1 |
| C13 | D14 | −0.29 | 1 |
| C14 | D14 | −0.14 | 1 |
| C21 | D14 | −0.14 | 1 |
| C22 | D14 | −0.36 | 1 |
| C23 | D14 | −0.22 | 1 |
| C24 | D14 | −0.22 | 1 |
| C31 | D14 | −0.29 | 1 |
| C32 | D14 | −0.22 | 1 |
| C33 | D14 | −0.29 | 1 |
| C34 | D14 | 0.67 | 1 |
| D12 | D14 | −0.12 | 1 |
| D13 | D14 | −0.19 | 1 |
| B311 | D21 | 0.57 | 1 |
| B312 | D21 | −0.12 | 1 |
| B313 | D21 | 0.41 | 1 |
| B314 | D21 | 0.5 | 1 |
| B321 | D21 | −0.12 | 1 |
| B322 | D21 | −0.25 | 1 |
| B323 | D21 | −0.25 | 1 |
| B331 | D21 | −0.12 | 1 |
| B332 | D21 | 0.5 | 1 |
| B333 | D21 | −0.12 | 1 |
| C11 | D21 | −0.14 | 1 |
| C12 | D21 | −0.28 | 1 |
| C13 | D21 | −0.29 | 1 |
| C14 | D21 | −0.14 | 1 |
| C21 | D21 | −0.14 | 1 |
| C22 | D21 | 0.45 | 1 |
| C23 | D21 | −0.22 | 1 |
| C24 | D21 | −0.22 | 1 |
| C31 | D21 | 0.38 | 1 |
| C32 | D21 | −0.22 | 1 |
| C33 | D21 | 0.38 | 1 |
| C34 | D21 | −0.29 | 1 |
| D12 | D21 | −0.12 | 1 |
| D13 | D21 | −0.19 | 1 |
| D14 | D21 | −0.12 | 1 |
| B311 | D22 | −0.37 | 1 |
| B312 | D22 | 0.66 | 1 |
| B313 | D22 | 0.31 | 1 |
| B314 | D22 | 0.19 | 1 |
| B321 | D22 | −0.19 | 1 |
| B322 | D22 | 0.8 | 1 |
| B323 | D22 | 0.31 | 1 |
| B331 | D22 | −0.19 | 1 |
| B332 | D22 | 0.76 | 1 |
| B333 | D22 | −0.19 | 1 |
| C11 | D22 | 0.65 | 1 |
| C12 | D22 | 0.87 | 1 |
| C13 | D22 | 0.15 | 1 |
| C14 | D22 | −0.22 | 1 |
| C21 | D22 | −0.22 | 1 |
| C22 | D22 | 0.69 | 1 |
| C23 | D22 | 0.33 | 1 |
| C24 | D22 | 0.33 | 1 |
| C31 | D22 | −0.44 | 1 |
| C32 | D22 | 0.33 | 1 |
| C33 | D22 | 0.29 | 1 |
| C34 | D22 | 0.073 | 1 |
| D12 | D22 | 0.66 | 1 |
| D13 | D22 | 0.43 | 1 |
| D14 | D22 | −0.19 | 1 |
| D21 | D22 | −0.19 | 1 |
| B311 | D23 | −0.25 | 1 |
| B313 | D23 | 0.65 | 1 |
| B314 | D23 | −0.25 | 1 |
| B321 | D23 | −0.12 | 1 |
| B322 | D23 | 0.41 | 1 |
| B323 | D23 | 0.65 | 1 |
| B331 | D23 | −0.12 | 1 |
| B332 | D23 | 0.5 | 1 |
| B333 | D23 | −0.12 | 1 |
| C12 | D23 | 0.66 | 1 |
| C13 | D23 | 0.49 | 1 |
| C14 | D23 | −0.14 | 1 |
| C21 | D23 | −0.14 | 1 |
| C22 | D23 | 0.45 | 1 |
| C23 | D23 | 0.65 | 1 |
| C24 | D23 | −0.22 | 1 |
| C31 | D23 | −0.29 | 1 |
| C32 | D23 | 0.65 | 1 |
| C33 | D23 | 0.67 | 1 |
| C34 | D23 | 0.38 | 1 |
| D13 | D23 | 0.75 | 1 |
| D14 | D23 | −0.12 | 1 |
| D21 | D23 | −0.12 | 1 |
| D22 | D23 | 0.66 | 1 |
| B311 | D24 | 0.33 | 1 |
| B312 | D24 | −0.12 | 1 |
| B313 | D24 | 0.41 | 1 |
| B314 | D24 | 0.5 | 1 |
| B321 | D24 | −0.12 | 1 |
| B322 | D24 | 0.41 | 1 |
| B323 | D24 | 0.41 | 1 |
| B331 | D24 | −0.12 | 1 |
| B332 | D24 | −0.25 | 1 |
| C11 | D24 | −0.14 | 1 |
| C12 | D24 | 0.28 | 1 |
| C13 | D24 | 0.49 | 1 |
| C22 | D24 | 0.09 | 1 |
| C23 | D24 | 0.65 | 1 |
| C24 | D24 | 0.65 | 1 |
| C31 | D24 | 0.38 | 1 |
| C32 | D24 | 0.65 | 1 |
| C33 | D24 | 0.38 | 1 |
| C34 | D24 | 0.38 | 1 |
| D12 | D24 | −0.12 | 1 |
| D13 | D24 | 0.56 | 1 |
| D14 | D24 | −0.12 | 1 |
| D21 | D24 | −0.12 | 1 |
| D22 | D24 | −0.19 | 1 |
| D23 | D24 | −0.12 | 1 |
| B311 | D31 | −0.37 | 1 |
| B312 | D31 | 0.66 | 1 |
| B313 | D31 | 0.31 | 1 |
| B314 | D31 | 0.19 | 1 |
| B321 | D31 | −0.19 | 1 |
| B322 | D31 | 0.8 | 1 |
| B323 | D31 | 0.31 | 1 |
| B331 | D31 | −0.19 | 1 |
| B332 | D31 | 0.76 | 1 |
| B333 | D31 | −0.19 | 1 |
| C11 | D31 | 0.65 | 1 |
| C12 | D31 | 0.87 | 1 |
| C13 | D31 | 0.15 | 1 |
| C14 | D31 | −0.22 | 1 |
| C21 | D31 | −0.22 | 1 |
| C22 | D31 | 0.69 | 1 |
| C23 | D31 | 0.33 | 1 |
| C24 | D31 | 0.33 | 1 |
| C31 | D31 | −0.44 | 1 |
| C32 | D31 | 0.33 | 1 |
| C33 | D31 | 0.29 | 1 |
| C34 | D31 | 0.073 | 1 |
| D12 | D31 | 0.66 | 1 |
| D13 | D31 | 0.43 | 1 |
| D14 | D31 | −0.19 | 1 |
| D21 | D31 | −0.19 | 1 |
| D23 | D31 | 0.66 | 1 |
| D24 | D31 | −0.19 | 1 |
| B311 | D32 | 0.57 | 1 |
| B312 | D32 | −0.12 | 1 |
| B313 | D32 | 0.41 | 1 |
| B314 | D32 | 0.5 | 1 |
| B321 | D32 | −0.12 | 1 |
| B322 | D32 | −0.25 | 1 |
| B323 | D32 | −0.25 | 1 |
| B331 | D32 | −0.12 | 1 |
| B332 | D32 | 0.5 | 1 |
| B333 | D32 | −0.12 | 1 |
| C11 | D32 | −0.14 | 1 |
| C12 | D32 | −0.28 | 1 |
| C13 | D32 | −0.29 | 1 |
| C14 | D32 | −0.14 | 1 |
| C21 | D32 | −0.14 | 1 |
| C22 | D32 | 0.45 | 1 |
| C23 | D32 | −0.22 | 1 |
| C24 | D32 | −0.22 | 1 |
| C31 | D32 | 0.38 | 1 |
| C32 | D32 | −0.22 | 1 |
| C33 | D32 | 0.38 | 1 |
| C34 | D32 | −0.29 | 1 |
| D12 | D32 | −0.12 | 1 |
| D13 | D32 | −0.19 | 1 |
| D14 | D32 | −0.12 | 1 |
| D22 | D32 | −0.19 | 1 |
| D23 | D32 | −0.12 | 1 |
| D24 | D32 | −0.12 | 1 |
| D31 | D32 | −0.19 | 1 |
| B311 | D34 | 0.47 | 1 |
| B312 | D34 | −0.25 | 1 |
| B313 | D34 | −0.10 | 1 |
| B314 | D34 | −0.055 | 1 |
| B321 | D34 | 0.41 | 1 |
| B322 | D34 | −0.48 | 1 |
| B323 | D34 | −0.48 | 1 |
| B331 | D34 | 0.41 | 1 |
| B332 | D34 | −0.055 | 1 |
| B333 | D34 | −0.25 | 1 |
| C11 | D34 | −0.29 | 1 |
| C12 | D34 | −0.57 | 1 |
| C13 | D34 | −0.59 | 1 |
| C14 | D34 | −0.29 | 1 |
| C21 | D34 | −0.29 | 1 |
| C22 | D34 | −0.25 | 1 |
| C23 | D34 | −0.44 | 1 |
| C24 | D34 | −0.44 | 1 |
| C31 | D34 | 0.37 | 1 |
| C32 | D34 | −0.44 | 1 |
| C33 | D34 | −0.18 | 1 |
| C34 | D34 | 0.22 | 1 |
| D12 | D34 | −0.25 | 1 |
| D13 | D34 | −0.37 | 1 |
| D14 | D34 | 0.65 | 1 |
| D21 | D34 | 0.41 | 1 |
| D22 | D34 | −0.37 | 1 |
| D23 | D34 | −0.25 | 1 |
| D24 | D34 | −0.25 | 1 |
| D31 | D34 | −0.37 | 1 |
| D32 | D34 | 0.41 | 1 |

**Supplementary Figure S1.** Comparison of *E. giganteana* moth weights among semiochemical treatments in the flight mill assay. There was no significant difference in weights of moths among any of the treatments tested.

**Supplementary Figure S2.** Representative mass spectra showing the *m*/*z* ratio against abundance from isolated *Silphium* samples (top row), a commercially purchased standard from Supelco (Terpene Mix A; middle row), and the relative abundance from NIST14 reference hits (bottom row). The compounds β-myrcene, β-phellandrene, β-pinene, camphene, and limonene were confirmed.

Supplementary Figure S3. Corrected antennal depolarization for the first (darker) and second (puff) of each treatment by female (orange) (n = 15 biological replicates per treatment) and male (blue) (n = 17 biological replicates per treatment) *E. giganteana* via EAG across the seven semiochemical treatments. Thick black horizontal bar represents median on each boxplot. Couplet boxplots with “ns” are not significantly different from each other (Tukey HSD, α = 0.05).
